# Health-related quality of life and quality-adjusted progression free survival for carfilzomib and dexamethasone maintenance following salvage autologous stem-cell transplantation in patients with multiple myeloma: a randomized phase 2 trial by the Nordic Myeloma Study Group

**DOI:** 10.1186/s41687-024-00691-2

**Published:** 2024-02-05

**Authors:** Lene Kongsgaard Nielsen, Fredrik Schjesvold, Sören Möller, Nina Guldbrandsen, Markus Hansson, Kari Remes, Valdas Peceliunas, Niels Abildgaard, Henrik Gregersen, Madeleine T. King

**Affiliations:** 1 Quality of life Research Center, Department of Hematology, Odense University Hospital, DK-5000 Odense, Denmark; 2 Department of Hematology, Gødstrup Hospital, Herning, Denmark; 3 Academy of Geriatric Cancer Research, Odense University Hospital, Odense, Denmark; 4 Oslo Myeloma Center, Department of Hematology, Oslo University Hospital, Oslo, Norway; 5 KG Jebsen Center for B cell malignancies, University of Oslo, Oslo, Norway; 6 Open Patient data Explorative Network, OPEN, Odense University Hospital, Odense, Denmark; 7 Department of Clinical Research, University of Southern Denmark, Odense, Denmark; 8 Department of Hematology, Skåne University Hospital, Lund, Sweden; 9 Department of Hematology, Turku University Hospital, Turku, Finland; 10 Department of Hematology, Vilnius University Hospital Santaros Klinikos, Vilnius, Lithuania; 11 Department of Hematology, Aalborg University Hospital, Aalborg, Denmark; 12 School of Psychology, University of Sydney, Sydney, Australia

**Keywords:** Randomized clinical trial, Multiple myeloma, Health-related quality of life, Carfilzomib

## Abstract

**Background:**

Decisions regarding maintenance therapy in patients with multiple myeloma should be based on both treatment efficacy and health-related quality of life (HRQL) consequences. In the CARFI trial, patients with first relapse of multiple myeloma underwent salvage autologous stem cell transplantation (salvage ASCT) before randomization to carfilzomib-dexamethasone maintenance therapy (Kd) or observation. The primary clinical endpoint was time to progression, which was extended by 8 months by Kd. The aim of this paper is to present the all HRQL endpoints of the CARFI trial including the HRQL effect of Kd maintenance therapy relative to observation. The primary HRQL endpoint was assessed by EORTC QLQ-C30 Summary score (QLQ-C30-sum) at 8 months follow-up. A key secondary HRQL endpoint was quality-adjusted progression-free-survival (QAPFS).

**Methods:**

HRQL was assessed with EORTC QLQ-C30, EORTC QLQ-MY20 and FACT/GOG-Ntx at randomization and every second month during follow-up. HRQL data were analyzed with linear mixed effect models until 8 months follow-up. QAPFS per individual was calculated by multiplying progression-free survival (PFS) by two quality-adjustment metrics, the QLQ-C30-sum and EORTC Quality of Life Utility Measure-Core 10 dimensions (QLU-C10D). The QAPFS per treatment group was estimated with the Kaplan-Meier method. *P* < 0.05 was used for statistical significance, and a between-group minimal important difference of 10 points was interpreted as clinically relevant for the QLQ-C30-sum.

**Results:**

168 patients were randomized. HRQL questionnaire compliance was 93%. For the QLQ-C30-sum, the difference of 4.62 points (95% confidence interval (CI) -8.9: -0.4, *p* = 0.032) was not clinically relevant. PFS was 19.3 months for the Kd maintenance group and 16.8 months for the observation group; difference = 2.5 months (95% CI 0.5; 4.5). QAPFS based on the QLQ-C30-sum for the Kd maintenance group was 18.0 months (95% CI 16.4; 19.6) and for the observation group 15.0 months (95% CI 13.5; 16.5); difference = 3.0 months (95% CI 0.8–5.3). QAPFS based on the QLU-C10D for the Kd maintenance group was 17.5 months (95% CI 15.9; 19.2) and 14.0 months (95% CI 12.4; 15.5) for the observation group; difference = 3.5 months (95% CI 1.1–5.9).

**Conclusions:**

Kd maintenance therapy after salvage ASCT did not adversely affect overall HRQL, but adjustment for HRQL reduced the PFS compared to unadjusted PFS. PFS of maintenance therapy should be quality-adjusted to balance the benefits and HRQL impact.

**Supplementary Information:**

The online version contains supplementary material available at 10.1186/s41687-024-00691-2.

## Background

Primary endpoints in clinical cancer trials are preferably objective, well-defined and measurable outcomes, such as overall survival, progression free survival and response rates [1]. However, secondary or exploratory endpoints, such as health-related quality of life (HRQL) measured through patient-reported outcomes (PROs), have become increasingly important to supplement the overall study findings [2]. PROs provide a patient-focused assessment of the impact of a treatment on patients´ symptoms and functional abilities, which can inform regulatory label claims and clinical decision making [3–5].

Multiple myeloma (MM) is an incurable hematological cancer associated with bone destruction, hypercalcemia, anemia, renal failure and infections [6]. Overall survival has markedly increased after the introduction of novel treatments in the past two decades [7–9]. A contributor to improved overall survival is high-dose melphalan with autologous stem-cell transplantation (ASCT), which is standard treatment in patients with newly diagnosed MM younger than 70 years of age without significant comorbidities [10, 11]. High-dose melphalan causes acute toxicity of anorexia, mucositis with pain and diarrhea, neutropenia and thrombocytopenia with temporary impairment of HRQL and full recovery for some patients, 1–2 months post-ASCT, while other patients still have moderate to severe symptoms one year post-ASCT [12–15].

Another contributor to improved overall survival in MM is maintenance treatment, i.e. long term therapy that extends response duration [16–18]. An important consideration for individual decision-making about maintenance therapy is the long-term impact on patient HRQL [17, 19]. Results from secondary PRO endpoints have shown that HRQL during maintenance varies with the specific drug used. Despite thalidomide´s prolongation of progression free survival (PFS), this drug is not approved for maintenance therapy for MM due to unacceptable impairment of HRQL [20, 21]. Lenalidomide maintenance improves PFS and overall survival and is approved for maintenance therapy in transplant-eligible patients after ASCT [18]. Adverse event registration from clinical trials shows that lenalidomide maintenance after ASCT is tolerable, and includes mainly hematological side-effects, diarrhea and fatigue [22–24]. However, randomized studies comparing HRQL during lenalidomide maintenance versus observation after ASCT are lacking. Published studies allowed several drugs as maintenance therapy or were designed with another drug as comparator [25–27]. In one of the studies, HRQL comparison between patients receiving maintenance therapy versus no maintenance therapy after first-line ASCT was done [27]. Several maintenance drugs were included, but not carfilzomib. The overall results indicated minimal impact on HRQL by maintenance therapy, but with worsening diarrhea and reduction in future perspectives.

Eventually, almost all patients with MM will experience relapse or progressive disease and for these patients, salvage ASCT will be an option, particularly for patients who achieved a long remission after frontline ASCT [10, 28]. After successful salvage ASCT, it would be desirable to prolong response duration with maintenance therapy, as long as that does not compromise HRQL or hamper recovery after salvage ASCT. However, there is limited randomized evidence on maintenance therapy after salvage ASCT. In the ReLApsE trial, lenalidomide maintenance therapy was included in the transplant arm; but the study was designed to evaluate salvage ASCT, not maintenance therapy [29].

To investigate the impact of maintenance therapy after salvage ASCT, the Nordic Myeloma Study Group initiated the CARFI trial in 2015 [30]. Carfilzomib, a second-generation proteasome inhibitor, was chosen for maintenance therapy in the CARFI trial, administered with dexamethasone every second week. The primary clinical endpoint of the CARFI study was time to progression; as previously reported, time to progression was significantly prolonged by eight months for the carfilzomib-dexamethasone (Kd) maintenance group (25.1 months) compared to observation (16.7 months) [30]. HRQL was one of the secondary endpoints of the CARFI trial; results of the European Organisation for Research and Treatment of Cancer (EORTC) Quality of Life Questionnaire– Core 30 items (QLQ-C30) global health status/quality of life (GHS/QoL) subscale showed no between-group difference during the 2-year follow-up period [30]. The aim of this paper is to present an in-depth analysis of the CARFI trial PRO endpoints, as per the statistical analysis plan [31].

### PRO objective, endpoints and hypotheses

The objective of the HRQL component of the CARFI trial was to assess the impact of Kd maintenance therapy, relative to observation, post salvage ASCT on a range of relevant aspects of HRQL. The primary PRO endpoint was change in overall HRQL from randomization to eight months follow-up, with the corresponding primary PRO hypothesis that there was no difference between the Kd maintenance therapy and observation groups. The primary PRO endpoint was augmented by the average overall HRQL per-patient based on all completed HRQL forms from randomization to last follow-up time-point prior to progressive disease/death/drug discontinuation/end of study (whichever came first). These two primary endpoints were supplemented by secondary PRO endpoints defined in terms of specific HRQL domains from randomization to 8 months later: (1) mean change; (2) the proportion of patients who improved, remained stable or worsened; (3) time to first recorded improvement; and (4) the proportion of patients with Kd-related symptoms. The final secondary endpoint was quality-adjusted progression free survival (QAPFS) from randomization to progressive disease/death/drug discontinuation/end of study (whichever came first). Hypotheses corresponding to each of these endpoints, including specific HRQL domains and symptoms, the questionnaires used to assess these, and the analysis and interpretation approach are presented in Table 1.

**Table 1:** Health-related quality of life endpoints, hypotheses, analysis and interpretation approach of the CARFI trial

Endpoints	HRQLdomains and/or items^1^	Hypotheses	Analysis and interpretation approach
**Primary endpoint, primary analysis**			
Change in overall HRQL from randomization to eight months follow-up.	EORTC QLQ-C30 Summary Score	There will be no difference between the two groups^2^ in change from randomization to eight months follow-up.	Non-inferiority approach. Between-group difference in mean change per group estimated using linear mixed effect model for repeated measures. *P*-value < 0.01 defined as statistically significant. MID > 10 defined as clinically relevant, used as the agreement-limit for non-inferiority, i.e. an estimated between-group difference less than 10 points would accept the non-inferiority hypothesis. Missing data handled as missing at random.
***Supportive analysis of primary endpoint***			
Average per-patient overall HRQL from randomization to progressive disease/death/drug discontinuation/end of study (whichever came first).	EORTC QLQ-C30 Summary Score	There will be no difference between the two groups^2^ in per-patient mean overall HRQL averaged across all available time points from randomization to progressive disease/death/drug discontinuation/end of study (whichever came first).	Non-inferiority approach comparing the mean of the average per-patient QLQ-C30-sum score between the two groups with the rank-sum test. *P*-value < 0.01 defined as statistically significant. MID > 10 used as the agreement-limit for non-inferiority, i.e. an estimated between-group difference less than 10 points would accept the non-inferiority hypothesis. Missing data handled as missing not at random.
***Secondary endpoints***			
1. Change in HRQL from randomization to eight months follow-up.	All individual HRQL domains of EORTC QLQ-C30 and QLQ-MY20, and FACT/GOG-ntx instruments	There will be no difference between the two groups^2^ in HRQL, except for the domains potentially impacted by Kd-related side effects (nausea/ vomiting, fatigue, dyspnea, diarrhea, body image). Even for these five domains, the differences will not exceed the threshold for clinically relevant differences.	Non-inferiority approach using linear mixed effect model for repeated measures. *P*-value < 0.05 defined as statistical significant. Evidence-based MID threshold used as the agreement-limit for non-inferiority of between group differences for EORTC QLQ-C30 and QLQ-MY20 domains (50, 52) and 4.4 for FACT/GOG-ntx. Missing data handled as missing at random.
2. The proportion of patients who have improved, remained stable and worsened in HRQL from randomization to eight months follow-up.	Physical, role, social and emotional functioning, GHS/QoL, body image and future perspectives	The proportion of patients who improved/remained stable/worsened will be similar between the two groups^2^.	Superiority approach in favor of the observation group. Patient-level analysis using the responder definition threshold of 20 points for multi item domains and 33 points for body image for improvement or worsening. Chi-square test for between group comparisons. *P*-value < 0.05 defined as statistical significant. Missing data handled as missing completely at random.
3. The time to first recorded improvement in HRQL from randomization to eight months follow-up.	Physical, role, social and emotional functioning; GHS/QoL, body image and future perspectives	Patients randomized to Kd maintenance therapy will take longer time to achieve improvement in emotional functioning, role functioning and social functioning as well as in the body image domain, compared to observation, but will not differ in time to improve in physical functioning and GHS/QoL.	Superiority approach in favor of the observation group for all domains except physical functioning and GHS/QoL. Patient-level analysis to assess the first time of improvement defined as 20 points for the multi item domains and 33 points for body image compared to randomization. Proportional hazards Cox regression model was used to compare between groups. *P*-value < 0.05 defined as statistical significant. Missing data were handled as missing completely at random.
4. The proportion of patients with Kd-related symptoms and the proportion of patients with moderate to severe Kd-related symptoms from randomization to eight months follow-up.	From QLQ-C30: Fatigue, nausea/vomiting, dyspnoea, insomnia, diarrhoea. From QLQ-MY20: agitation/irritation.	A larger proportion of patients randomized to Kd maintenance therapy will report more severe levels of symptoms compared to observation.	Superiority approach in favor of the observation group. Patient-level analysis using the raw score thresholds for change from randomization of ≥ 33 points for Kd-related symptoms and ≥ 66 points for severe Kd-related symptoms. Chi-square test for between group comparisons. *P*-value < 0.05 defined as statistical significant. Missing data handled as missing completely at random.
5. Quality-adjusted progression-free survival from randomization to last follow-up time-point.	Two quality-adjustment metrics: EORTC QLQ-C30 Summary Score and EORTC QLU-C10D	Quality-adjusted progression-free survival will not differ substantially from the difference observed in progression free survival, since we do not expect Kd to impact significantly on overall HRQL.	Non-inferiority approach. Per-patient time in QAPFS calculation for each of the metrics and multiplied with the per-patient estimated mean time to last follow-up time point. Group-level QAPFS estimated by Kaplan-Meier method and difference between group were estimated using bootstrap methods. *P*-value < 0.05 defined as statistical significant. Missing data handled as missing completely at random.

HRQL; health-related quality of life, EORTC QLQ-C30; European Organisation for Research and Treatment of Cancer Quality of Life Questionnaire– Core 30 items, QLQ-MY20; the Multiple Myeloma module, FACT/GOG-ntx; Functional Assessment of cancer Therapy/Gynaecologic Oncolocy Group-Neurotoxity instrument, GHS/QoL; global health status/quality of life, QLU-C10D; the EORTC Quality of Life Utility Measure-Core 10 dimensions, QLQ-C30-sum; EORTC QLQ-C30 Summary, Kd; carfilzomib-dexamethasone, MID; minimal important difference, QAPFS; quality-adjusted progression-free survival

^1^As assessed by items and standard scoring algorithms of the EORTC QLQ-C30, EORTC QLQ-MY20 and FACT/GOG-ntx.

^2^The two groups refer to the Kd maintenance therapy group and the observation group

## Methods

### Design, patients and treatment

The CARFI trial was a Nordic Myeloma Study Group open-label multi-center, randomized, phase II clinical trial, using a parallel (1:1) group design (clinicaltrials.gov: NCT02572492). Details of the study design, trial treatment and main findings have been published elsewhere [30]. In brief, patients with MM aged 18 years or more with first relapse after prior ASCT and found eligible for salvage ASCT were included at 25 hospitals within four Nordic countries and Lithuania from January 2015 to April 2018. Key exclusion criteria were previous treatment with carfilzomib, maintenance treatment given after first ASCT, World Health Organization performance status ≥ 3, significant neuropathy (grade 3–4, or grade 2 with pain) and any comorbidity that would preclude treatment with carfilzomib or salvage ASCT.

Two months after reinduction with four cycles of carfilzomib-lenalidomide-dexamethasone followed by salvage ASCT, patients were randomized to Kd maintenance or observation. Kd maintenance was given as intravenous carfilzomib (27 mg/sqm every second week with escalation of carfilzomib to 56 mg/sqm if tolerated) and oral dexamethasone 20 mg every second week until progression, unacceptable adverse effects, withdrawal of consent or 1 September 2019 (end of study). Dose modifications of carfilzomib and/or dexamethasone were done in case of toxicity related to the drugs (described in the supplementary appendix).

### PRO instruments

HRQL were assessed with three instruments: the EORTC core module (QLQ-C30) [32, 33] and Multiple Myeloma module (QLQ-MY20) [34], and the Functional Assessment of cancer Therapy/Gynaecologic Oncolocy Group-Neurotoxity (FACT/GOG-ntx) instruments [35]. The three questionnaires and domains are described in Table 2.

**Table 2:** The three questionnaires, domains, descriptions and scoring. EORTC QLQ-C30; quality of life questionnaire-core 30, EORTC QLQ-MY20; quality of life questionnaire-multiple myeloma module, FACT/GOG-Ntx; Functional Assessment of cancer Therapy/Gynaecologic Oncology Group-Neurotoxicity

Health-related quality of life questionnaires	Domains	Description and scoring
EORTC QLQ-C30 (30)	Global quality of life Five functional domains (physical, role, emotional, cognitive and social) Nine symptom domains (fatigue, nausea and vomiting, pain, dyspnoea, insomnia, appetite loss, constipation, diarrhea) Financial difficulties	A 30-item, 15 domain cancer-generic questionnaire validated in patients with multiple myeloma (31). Recall period of 7 days. Four-point categorical scale: ‘not at all’, ‘a little’, ‘quite a bit’, ‘very much’. The answers are transformed into 0-100 scales (44). For the functional domains, a high score means low degree of problems. For the symptom domains, a high score means high degree of symptoms.
EORTC QLQ-MY20 (32)	Two functional domains (future perspective and body image) Two symptom domains (disease symptoms and side effects of treatment)	A 20-item, four domain myeloma-specific questionnaire. Recall period of 7 days. Four-point categorical scale: ‘not at all’, ‘a little’, ‘quite a bit’, ‘very much’. The answers are transformed into 0-100 scales (44). For the functional domains, a high score means low degree of problems. For the symptom domains, a high score means high degree of symptoms.
FACT/GOG-ntx (33)	Peripheral neuropathy	An 11-item questionnaire summarized as a single domain of peripheral neuropathy. Recall period of 7 days. Five-point categorical scale: ‘not at all’, ‘a little bit’, ‘somewhat’, ‘quite a bit’, ‘very much’. The answers are transformed into a 0–44 scale (45). Higher score indicating less peripheral neuropathy

### PRO data collection

Patient inclusion in the PRO sub-protocol was part of the CARFI trial. HRQL was assessed with all three instruments at randomization (two months post salvage ASCT) and then every second month in the outpatient clinic at study visits. The study nurses had access to trial specific guidance in PRO data collection. If a scheduled visit was postponed (e.g. due to vacation, public holidays, acute toxicities beyond grade 2), the PRO data collection was postponed as well. From study initiation until July 2017, the study nurses administered all three PRO instruments on paper for the patients to complete in the clinic. From July 2017, electronic completion of the EORTC instruments via a tablet directly into a REDCap database became an option. Real-time monitoring of non-completion of instruments was not carried out. PRO data collection continued until end of study for each patient, defined as the time of disease progression, death, drug discontinuation or study termination (whichever came first). Study termination was defined as the last protocol visit for the last included patient, which was on 1 September 2019.

### Rationale for PRO endpoints

In developing the statistical analysis plan, HRQL studies of patients with MM were reviewed to inform our choice of PRO endpoints and appropriate PRO measures [31]. The EORTC QLQ-C30 Summary (QLQ-C30-sum) score was chosen to assess the primary PRO endpoint because there is no standard HRQL assessment for use in MM maintenance trials, it captures the generic HRQL concepts of importance in cancer, it reduces issues with multiplicity (type I error), and it has been validated in patients with hematological malignancies [36, 37]. Secondary PRO endpoints were specified a priori, aligned with hypotheses (Table 1), assessed by specific domains from the QLQ-C30, QLQ-MY20 and FACT/GOG-ntx. These included four of the functioning domains (physical, role, social, emotional) and GHS/QoL from EORTC QLQ-C30, and two domains from the QLQ-MY20 (body image and future perspectives); these were chosen because they have previously been shown to be affected after primary ASCT [12–14]. Based on the literature, Kd-related symptoms were identified and defined as fatigue, dyspnea, nausea/vomiting, diarrhea, insomnia, agitation and restlessness; these were included to assess the non-infusion related symptomatic impact of carfilzomib and dexamethasone treatment [38–45]. Time in QAPFS was included as it captures the benefits and consequences of Kd maintenance therapy in terms of both treatment efficacy (PFS) and HRQL consequences. Further details are provided in the statistical analysis plan [31].

### Statistical analyses

The statistical analysis plan for the PRO endpoints was finalized and published before starting the PRO data analyses [31]; a clarifying amendment was added to the published statistical analysis plan before finalizing the PRO data analyses. The statistical analyses are described here in brief. For each patient, the study period was from randomization to that patient’s last follow-up prior to progressive disease/death/drug discontinuation/end of study (which ever came first). The analyses were based on all available questionnaires from randomized patients on protocol until last follow-up time point except for the supportive analysis of the primary endpoint where all available questionnaires were included in the analysis. The last follow-up time point was defined as the time point *before* the number of patients on protocol in one of the groups became less than 15 patients. Analyses were carried out using Stata 17.

Patient characteristics and PRO scores at randomization were described with summary statistics (mean and 95% confidence intervals (CI)). PRO completion rates were calculated for each assessment time point as number of randomized patients with enough completed items to calculate the QLQ-C30-sum score as a proportion of patients on protocol at that time point.

HRQL domain scores of the QLQ-C30 and QLQ-MY20 were calculated according to EORTC standard scoring [46]. The QLQ-C30-sum score was calculated from the mean of 13 of the 15 QLQ-C30 domains (excluding GHS/QoL and Financial impact defining items) [37]. The QLQ-C30-sum score ranges from 0to 100, with 100 being best. The FACT/GOG-ntx score was calculated according to the FACT manual [47].

For the endpoints involving change in HRQL, the longitudinal PRO data were analyzed with a linear mixed effect model for repeated measures. The model included patients as a random variable and time (baseline, two, four, six and eight months), treatment group and country as fixed factors. The results were tested for statistical significance at the 5% level for the primary HRQL endpoint and at the 1% level for the secondary HRQL endpoints. Statistically significant results were interpreted as clinically relevant/meaningful if they exceed the threshold for minimally important difference/change (MID). As MID has not yet been established for the QLQ-C30-sum score, a 10-point change/difference was pre-specified as indicating a clinically relevant change (within a group) and a clinically relevant difference in change between groups [48, 49]. Within and between group changes in the individual domains of the QLQ-C30 questionnaire were interpreted according to the evidence-based guidelines for between-group differences [50] and change over time [51] using the threshold for a small difference/change. For the domains of QLQ-MY20, recommended estimates for MIDs were used (disease symptoms and side effects of treatment; 10 points, body image; 13 points and future perspectives: 9 points) [52]. For the FACT/GOG-ntx subscale, 4.4 [53] points were used as primary MID and 11.8 points were used for sensitivity analysis [54].

For the supportive analysis of the primary endpoint, the average per-patient QLQ-C30-sum score from randomization to progressive disease/death/drug discontinuation/end of study was calculated. The mean per-patient QLQ-C30-sum score was calculated for each group and compared with a rank-sum test.

To evaluate the proportion of patients who improved, remained stable or worsened in HRQL, patients were categorized as having perceived either an improvement or worsening from randomization if they experienced a change in score (in the direction of improvement or worsening, respectively) that exceeded at least 20 points [55]. For the single item domain of body image, 33 points were used [52].

Time to first recorded improvement in HRQL was calculated for each patient as time from randomization to the first time the patient reported an improvement of at least 20 points for the EORTC multi-item domains [55], and 33 points for the body image single item domain [52]. Patients who did not record an improvement at any time point were censored at end of study. Patients with no score at randomization and patients with high functioning as well as good GHS/QoL (greater than 80 points) and body image (less than 33 points) at randomization were excluded from this analysis since this left no room for improvement. The mean time to improvement was compared between groups using a proportional hazards Cox regression model with treatment, PRO score at randomization and country as covariates, and presented as a hazard ratio (HR) with corresponding 95% CI. A post-hoc analysis of average time of first recorded improvement with corresponding standard deviations was performed.

For endpoints involving the proportion of patients with Kd-related symptoms, raw item scores were converted to 0-100 scales according to the EORTC manual [46], i.e. “none at all”=0, “a little”=33, “quite a bit”=67, “very much”=100. The proportion of patients with Kd-related symptoms was defined as the number of patients experiencing any of the Kd-related symptoms corresponding to at least “a little” (≥ 33 points) divided by the total number of patients. Similarly for the proportion of patients reporting moderate to severe Kd-related symptoms, a score of at least “quite a bit” (≥ 66 points) was used. This dichotomized grading has previously been used in patients with hematological malignancies [56]. Differences between treatment groups in these proportions were analyzed as binary endpoints using chi-square tests of homogeneity.

Preference-based utility scores were calculated from the QLQ-C30 data using the utility scoring algorithm of EORTC Quality of Life Utility Measure-Core 10 dimensions (QLU-C10D) [57], which yields a score with a maximum of one (representing the best possible health state), and is anchored at zero (representing the state of being dead). Implementing this algorithm requires a country-specific value set; as a Danish QLU-C10D value set was not available at the time of analysis, the German value set was used [58], as it was considered to be closest culturally of the country-specific value sets available at the time of analysis. The time in QAPFS was calculated using two quality adjustment metrics: the per-patient mean EORTC QLQ-C30-sum score (divided by 100, as required for quality adjustment of life years) and the per-patient mean QLU-C10D utility score [59]. Patient-level QAPFS was calculated by multiplying the per-patient estimated mean time to progression/death (censored at last follow-up time point) by each of the quality adjustment metrics. Group-level QAPFS was then estimated with the Kaplan-Meier method. The mean difference in QAPFS between the two groups was estimated using bootstrap methods for each quality-adjustment metric.

To examine potentially missing data patterns (informative drop-out) two pattern mixture model analyses were performed; (1) stratifying patients into two groups based on drop-out time early (drop-out between zero and eight months follow-up) and late (participated in the study at 10 months) per treatment group, (2) stratifying patients into three groups based on drop-out time (early, late and never drop-out) per treatment group.

A post-hoc analysis was performed to investigate compliance with Kd maintenance therapy by assessing the proportion of fully administered, reduced and omitted doses of carfilzomib and dexamethasone relative to scheduled doses.

Sample size in the maintenance phase of the CARFI trial was determined according to the primary trial endpoint (time to progression). According to the trial protocol, it was expected that 150 patients would continue to the maintenance phase. No formal power analysis was performed for the PROs, and no patients were involved in the design or the interpretation phase of the study.

## Results

### Patient population

**Fig. 1:**
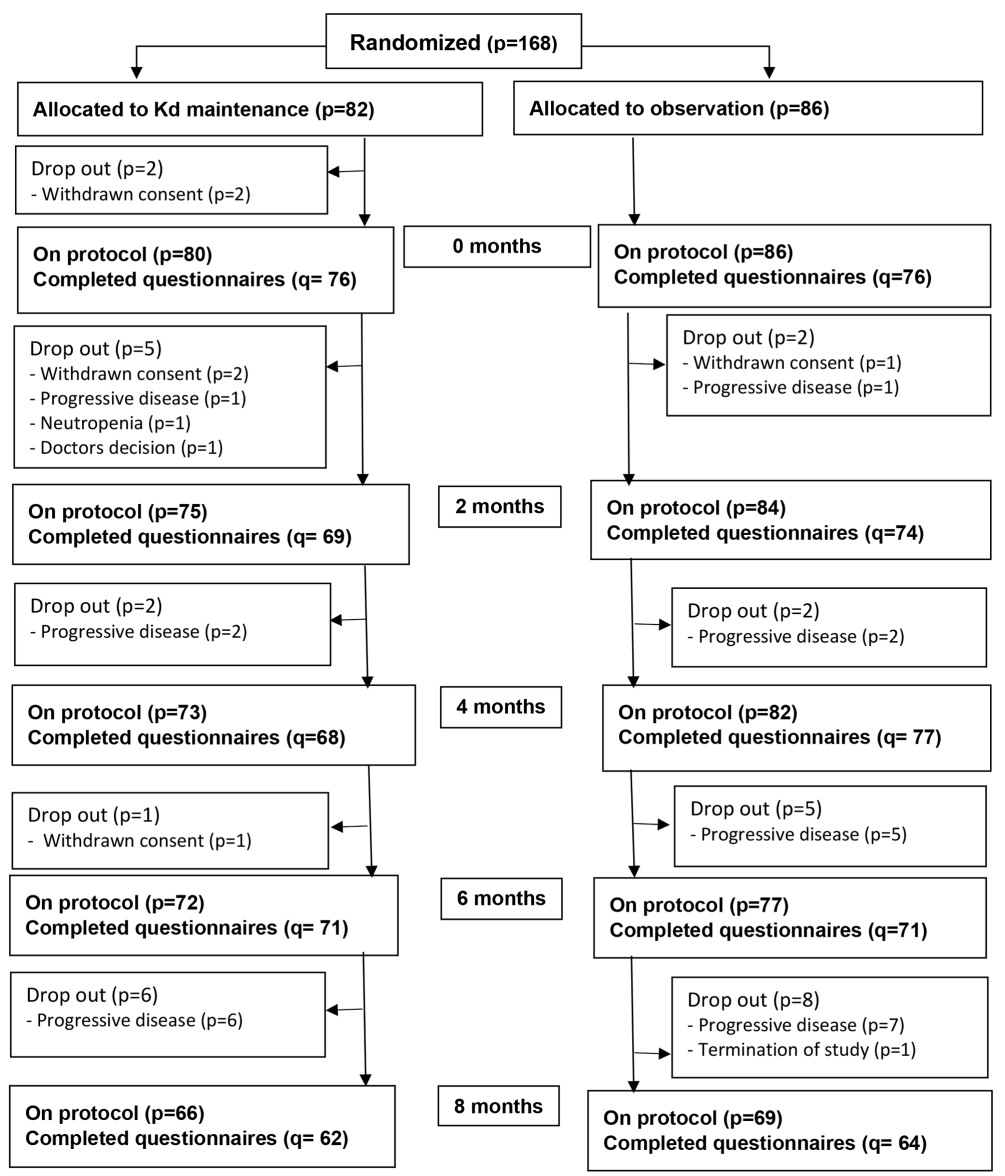
CONSORT flow diagram CONSORT flow diagram of the entire study period is presented in the supplementary appendix Figure S1 Kd; Carfilzomib-dexamethasone, p; patient, q; questionnaires (completed items for calculating the EORTC QLQ-C30 summary score)

In total, 181 patients underwent salvage ASCT, and 168 patients continued to randomization between Kd maintenance or observation; 82 were randomized to Kd maintenance and 86 to observation, constituting the population for this HRQL analysis. Reasons for drop-out before randomization have been reported previously [30]. Reasons for drop-out from randomization until eight months follow-up are presented in the CONSORT flow diagram of Fig. 1 and the supplementary appendix Figure S1 until last follow-up time point (i.e. 22 months). Patient demographics, clinical characteristics and PRO mean scores at randomization are presented in Table 3. PRO completion rate until eight months follow-up was 93% (95% for the Kd maintenance and 91% for the observation group) and 93% until last follow-up time point (supplementary appendix Table S1).

**Table 3:** Patient characteristics and patient-reported outcomes scores at randomization. Kd; Carfilzomib-dexamethsone, IQR; Interquartile range 25-75%, MM; multiple myeloma, ASCT; high-dose melphalan with autologous stem-cell transplantation, WHO; World health organization, EORTC QLQ-C30, European Organisation for Research and Treatment of Cancer Quality of Life Questionnaire core questionnaire; EORTC QLQ‐MY20, European Organisation for Research and Treatment of Cancer Multiple Myeloma module; FACT/GOG‐ntx subscale, Functional Assessment of Cancer Therapy/Gynecologic Oncology Group Neurotoxicity subscale; SD, Standard deviation

	Kd maintenance group (*p* = 82)	Observation group (*p* = 86)
**Median age**, years (IQR)	60 (54–65)	63 (58–67)
**Gender** Female/Male	39/43	32/54
**Median time from MM diagnosis**, months (IQR)	47 (37–65)	48 (37–60)
**Median time from salvage ASCT to randomization**, weeks (IQR)	8.1 (7.7–8.5)	8.1 (7.8–8.8)
**Median time from salvage ASCT to start of Kd maintenance/observation**, weeks (IQR)	9.8 (8.8–10.3)	10.8 (8.8–13.7)
**Myeloma status** Stringent complete response Complete response Very good partial response Partial response Stable disease Progressive disease Missing	10 (12%) 13 (16%) 41 (50%) 16 (20%) 1 (1%) 0 (0%) 1 (1%)	12 (14%) 8 (9%) 50 (58%) 16 (19%) 0 (0%) 0 (0%) 0 (0%)
**WHO performance status** 0 1 2 3 or 4 Missing	47 (57%) 29 (35%) 2 (2%) 0 (0%) 4 (5%)	45 (52%) 30 (35%) 2 (2%) 0 (0%) 9 (10%)
**Country of residence** Denmark Sweden Norway Finland Lithuanian	26 (30%) 18 (21%) 32 (37%) 4 (5%) 6 (7%)	21 (26%) 28 (34%) 20 (24%) 4 (5%) 9 (11%)
**EORTC QLQ-C30 domain scores, mean (SD)**		
Global Health Status/Quality of life	66.0 (22.3)	68.1 (18.8)
Physical functioning	77.4 (19.9)	75.9 (20.0)
Role functioning	65.6 (29.5)	63.0 (29.6)
Emotional functioning	80.1 (21.3)	85.8 (17.9)
Cognitive functioning	84.8 (18.3)	82.3 (21.7)
Social functioning	74.1 (24.6)	73.6 (25.4)
Fatigue	35.6 (24.2)	35.6 (21.4)
Nausea/vomiting	7.7 (14.4)	9.0 (18.9)
Pain	22.9 (25.4)	25.2 (27.8)
Dyspnea	23.2 (25.0)	25.6 (25.7)
Insomnia	23.5 (26.4)	23.5 (25.8)
Appetite loss	13.0 (21.7)	16.2 (25.6)
Constipation	9.0 (19.1)	7.8 (19.4)
Diarrhea	17.9 (24.4)	19.7 (26.8)
Financial difficulties	16.2 (26.7)	8.6 (21.9)
**EORTC QLQ-MY20 domain scores, mean (SD)**		
Disease symptoms	17.5 (16.9)	17.3 (17.5)
Side effects of treatment	22.2 (14.2)	20.2 (15.2)
Future perspective	58.9 (29.1)	62.0 (25.9)
Body image	68.4 (30.3)	70.2 (32.7)
**FACT/GOG-ntx domain score, mean (SD)**		
FACT/GOG-ntx subscale	38.1 (6.7)	38.7 (5.0)

### Effect of kd maintenance on HRQL

**Fig. 2:**
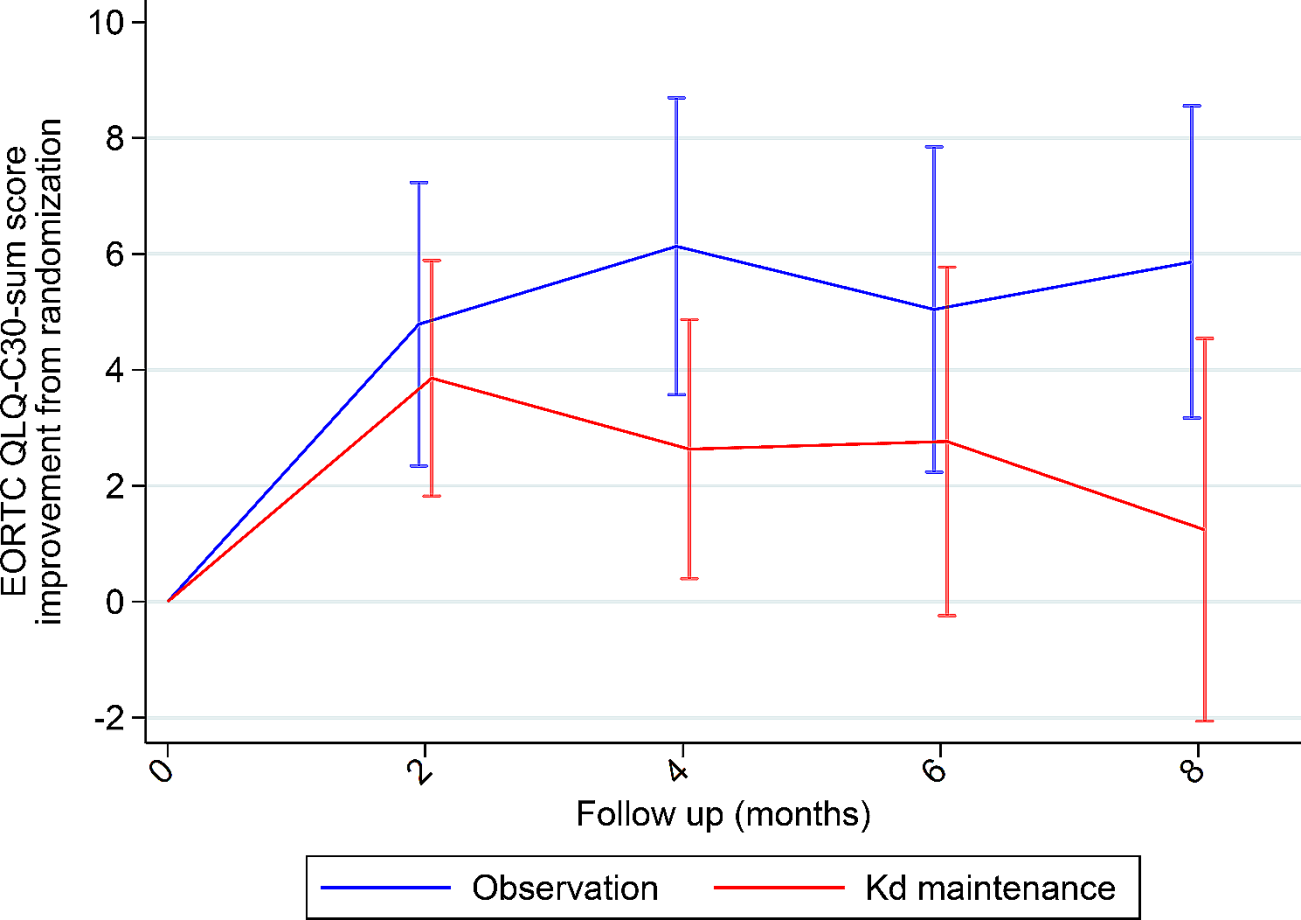
Mean score change from randomization in EORTC QLQ-C30 Summary Score until 8 months follow-up. Horizontal bars illustrates 95% confidence intervals. Kd; Carfilzomib-dexamethasone

The difference in mean change from randomization between groups in QLQ-C30-sum score estimated at eight months was 4.62 points on the 0-100 scale (95% CI -8.9; -0.4, *p* = 0.032) was statistically significant but not clinically important (i.e. it was less than the predefined MID). The findings are visualized in Fig. 2. Similarly, none of the differences at specific time points (two, four and six months follow-up) reached statistical significance and clinical relevance. Data until last follow-up time point are provided in the supplemental appendix Table S2. Results of the pre-specified supportive analysis of the primary endpoint, the average per-patient mean QLQ-C30-sum score, was 81.4 (SD 12.9) for the Kd maintenance group and 82.5 (SD 12.3) for the observation group. These means were similar and did not differ statistically significantly between the groups (rank sum test, *p* = 0.625).

### Effect of kd maintenance on the individual PRO domains

**Fig. 3:**
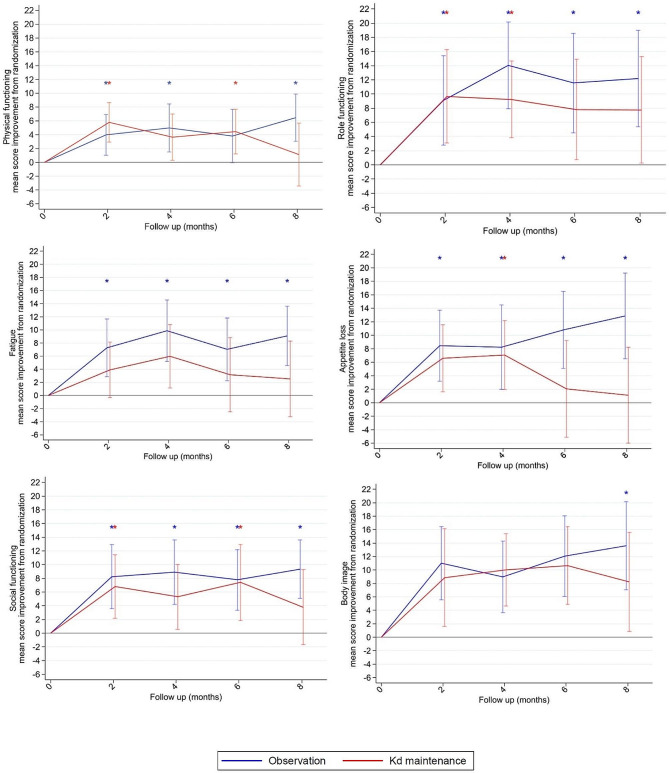
Mean score improvement from randomization to 8 months follow-up for the six domains with statistically significant and clinically meaningful improvement at 8 months follow-up Blue* indicates time points with statistically significant and clinically meaningful improvement for the observation group. Red* indicates time points with statistically significant and clinically meaningful improvement for the carfilzomib-dexamethasone (Kd) maintenance group

Within the observation group, the patients reported statistically significant and clinically meaningful improvements at eight months in six domains (physical, role, social functioning, appetite loss, fatigue and body image), whereas the patients in the Kd maintenance group did not report statistically significant or clinically meaningful improvements in any domains at eight months follow-up. The findings are presented in Table 4. For these six domains, the results at eight months are presented in Fig. 3, and mean score changes, 95% confidence intervals and *p*-values until last follow-up time point are presented in supplementary appendix Table S3-S8.

**Table 4:** Mean change from randomization to eight months follow-up and between-group difference. The point estimates in bold indicate a statistically significant (*p* < 0.01) and clinically relevant difference/meaningful change. Improvements are indicated by positive changes for function domains and negative changes for symptom domains

	Kd maintenance group		Observation only group		Between groups	
	Mean score change (95%CI)	*p*-value	Mean score change (95%CI)	*p*-value	Mean score difference (95%CI)	*p*-value
**QLQ-C30 function domains**	**Positive values indicate better functioning**				**Negative values favour observation group**	
GHS/QoL	1.77 (-5.14; 8.69)	0.615	1.36 (-3.71; 6.43)	0.599	0.41 (-8.16; 8.99)	0.925
Physical	1.13 (-3.43; 5.69)	0.628	**6.47 (3.05; 9.89)**	**< 0.001**	-5.34 (-10.94; 0.26)	0.062
Role	7.74 (0.23; 15.25)	0.043	**12.18 (5.39; 18.96)**	**< 0.001**	-4.44 (-14.52; 5.65)	0.389
Social	3.81 (-1.67; 9.28)	0.173	**9.34 (5.07; 13.62)**	**< 0.001**	-5.54 (-12.41; 1.34)	0.114
Emotional	0.57 (-3.14; 4.27)	0.765	0.00 (-3.35; 3.35)	0.999	0.56 (-4.44; 5.57)	0.826
Cognitive	-1.80 (-5.85; 2.25)	0.383	2.23 (-1.82; 6.28)	0.281	-4.03 (-9.79; 1.73)	0.170
**QLQ-C30 Symptom domains**	**Negative values indicate reduced symptoms**				**Positive values favor observation group**	
Appetite loss	-1.11 (-8.21; 5.99)	0.76	**-12.87 (-19.21;-6.52)**	**< 0.001**	11.75 (2.47; 21.04)	0.013
Constipation	-0.95 (-6.00; 4.11)	0.714	-0.86 (-5.54; 3.82)	0.719	-0.09 (-7.01; 6.84)	0.980
Diarrhoea	-0.86 (-7.72; 6.00)	0.806	-4.81 (-11.62; 2.01)	0.167	3.95 (-5.78; 13.67)	0.426
Dyspnoea	-1.23 (-7.68; 5.22)	0.709	-6.79 (-13.60; 0.01)	0.050	5.56 (-4.01; 15.14)	0.255
Fatigue	-2.53 (-8.30; 3.24)	0.390	**-9.09 (-13.61; -4.57)**	**< 0.001**	6.56 (-0.72; 13.84)	0.078
Nausea and vomiting	0.34 (-4.57; 5.26)	0.891	-5.91 (-10.59; -1.22)	0.013	6.25 (-0.31; 12.81)	0.062
Pain	1.73 (-4.04; 7.50)	0.557	-1.90 (-7.14; 3.33)	0.476	3.63 (-4.07; 11.34)	0.356
Insomnia	2.76 (-3.75; 9.27)	0.406	-2.70 (-8.50; 3.10)	0.362	5.46 (-3.19; 14.10)	0.216
Financial difficulties	-2.61 (-6.69; 1.47)	0.211	-0.15 (-3.13; 2.82)	0.920	-2.46 (-7.57; 2.66)	0.346
**QLQ-MY20 function domains**	**Positive values indicate better functioning**				**Negative values favor observation group**	
Future perspectives	7.64 (3.17; 12.11)	0.001	7.58 (3.16; 12.00)	0.001	0.06 (-6.33; 6.44)	0.986
Body image	8.24 (0.90; 15.58)	0.028	**13.60 (7.05; 20.14)**	**< 0.001**	-5.36 (-15.30; 4.59)	0.291
**QLQ-MY20 symptom domains**	**Negative values indicate reduced symptoms/side effects**				**Positive values favor observation group**	
Disease symptoms	3.07 (-0.20; 6.35)	0.066	0.72 (-2.54; 3.97)	0.666	2.36 (-2.23; 6.94)	0.314
Side effects of treatment	-5.21 (-8.53; -1.89)	0.002	-7.09 (-9.80; -4.38)	< 0.001	1.88 (-2.39; 6.16)	0.388
**FACT/GOG-Ntx subscale**	**Positive values indicate less peripheral neuropathy**				**Negative values favour observation group**	
FACT/GOG-Ntx subscale	-0.63 (-2.11-0.84)	0.399	0.85 (0.12–1.57)	0.022	-1.48 (-3.14-0.18)	0.080

Kd; Carfilzomib-dexamethasone, GHS/QoL; global health status/quality of life, QLQ-C30; quality of life questionnaire-core 30, QLQ-MY20; quality of life questionnaire-multiple myeloma module, FACT/GOG-Ntx; Functional Assessment of cancer Therapy/Gynaecologic Oncology Group-Neurotoxicity CI; Confidence interval

### Proportion of patients who improved, remained stable or worsened

Generally, the majority (range 52-93%) of patients in both groups remained stable from randomization to the eight months follow-up, with some notable exceptions. A higher proportion of patients in the Kd maintenance compared to the observation group (93% versus 81%) reported stable physical functioning at four months (odds ratio 2.99, 95% CI 1.01; 8.83, *p* = 0.040). Also, a higher proportion of patients in the Kd maintenance group compared to the observation group (13% versus 2%) reported worsening in social functioning at eight months (odds ratio 8.74, 95% CI 1.06; 72.2, *p* = 0.018). The proportions and odds ratios for all domains from randomization to two, four, six and eight months follow-up are reported in Supplementary Table S9.

### Time to first recorded improvement

There were no statistically significant between-group difference in time to first recorded improvement in any of the (EORTC QLQ-C30) functional domains, GHS/QoL scale, QLQ-MY20 body image domain or future perspectives (Table 5). The average weeks to first recorded improvement is displayed in the supplementary appendix Table S10).

**Table 5:** Time to first recorded improvement in the functional domains, Global Health Scale/Quality of Life and body image domains. Patients with no score at randomization and patients with high functioning as well as good GHS/QoL and body image at randomization were excluded from this analysis since high/good score at randomization leaves no room for improvement

	Kd maintenance group		Observation group		Hazard ratio (95%CI)	*P*-value
	Patients included in the analysis (n)	Improved n (%)	Patients included in the analysis (n)	Improved n (%)		
Global Health scale/QoL	52	24 (46)	46	17 (37)	1.42 (0.73–2.76)	0.30
Physical functioning	40	15 (38)	43	15 (35)	1.03 (0.47–2.26)	0.94
Role functioning	48	31 (65)	53	38 (72)	0.83 (0.49–1.40)	0.49
Emotional functioning	32	10 (31)	22	10 (45)	0.66 (0.25–1.74)	0.40
Social functioning	39	23 (59)	40	23 (58)	0.95 (0.51–1.78)	0.88
Body image	48	34 (71)	42	32 (76)	0.86 (0.51–1.45)	0.58
Future perspectives	59	30 (51)	62	32 (52)	0.91 (0.52–1.59)	0.74

Kd; Carfilzomib-dexamethasone, QoL; Quality of life

### Proportion of patients with Kd-related symptoms

**Fig. 4:**
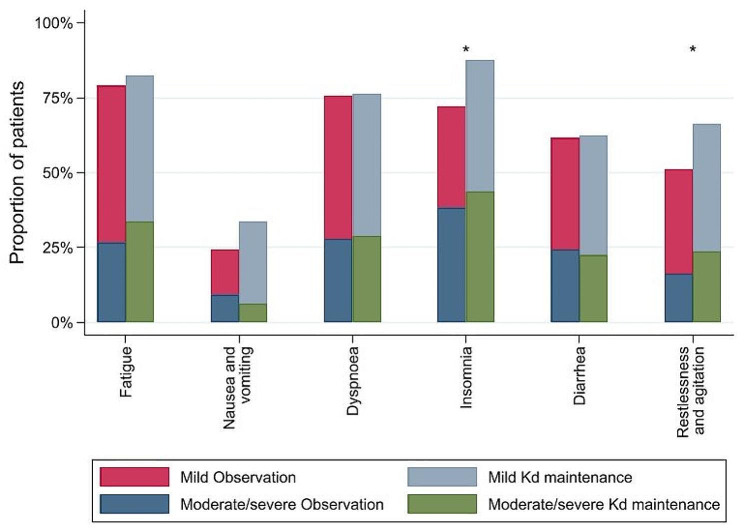
Proportion of patients developing carfilzomib-dexamethasone (Kd) related symptoms during the study period for the Kd maintenance and the observation group

Significantly more patients in the Kd maintenance group developed Kd-related symptoms of restlessness and agitation (odds ratio 1.90, CI95% 1.01; 3.58, *p* = 0.046) and insomnia (odds ratio 2.60, CI 95% 1.15; 5.88, *p* = 0.019) during the study period (Fig. 4 and the supplementary appendix Table S11).

### Quality-adjusted progression free survival

**Fig. 5:**
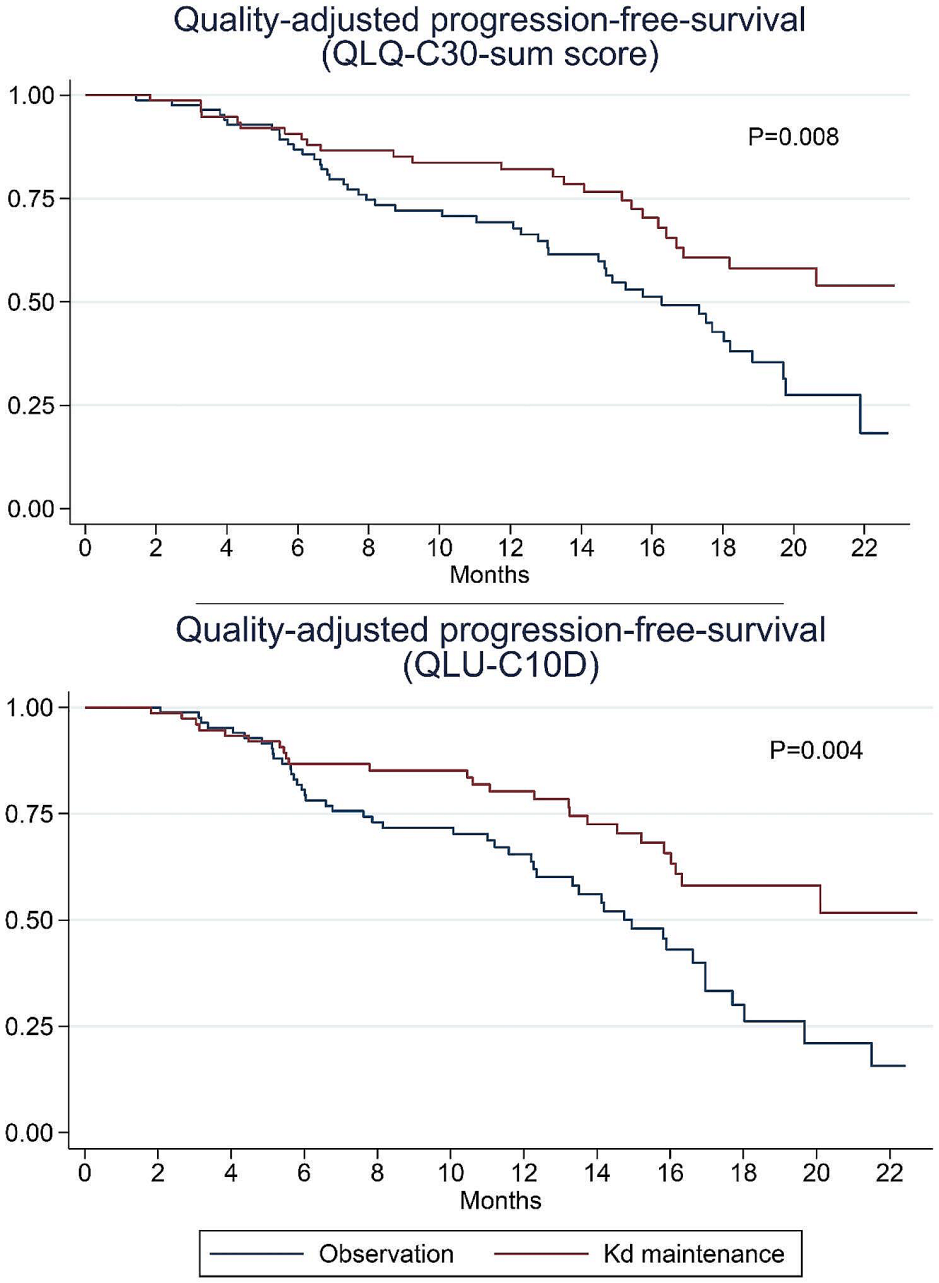
Quality-adjusted progression-free-survival based on the EORTC QLQ-C30 Summary score and EORTC Quality of Life Utility Measure-Core 10 dimensions (QLU-C10D). Kd; Carfilzomib-dexamethansone

When quality-adjusting the PFS based on the QLQ-C30-sum score, the QAPFS difference between the twogroups was 3.0 months in favor of the Kd maintenance group (95% CI 0.78; 5.28, *p* = 0.008). Mean QAPFS based on the QLQ-C30-sum score for the Kd maintenance group was 18.0 months (95% CI 16.4; 19.6) and 15.0 months for the observation group (95% CI 13.5; 16.5). Similarly for QAPFS based on the QLU-C10D, where Kd maintenance significantly extended QAPFS by 3.5 months (95% CI 1.2; 5.9, *p* = 0.004) compared to observation. The mean QAPFS based on the QLU-C10D for patients in the Kd maintenance group was 17.5 months (95% CI 15.9; 19.2) and 14.0 months (95% CI 12.4; 15.5) for the observation group. The quality-adjusted progression-free-survival curves are displayed in Fig. 5. For comparison, the unadjusted progression-free survival was 19.3 months for the Kd maintenance group (95% CI 17.9; 20.7) and 16.8 for the observation group (95% CI 15.4; 18.3) resulting in a significantly superior PFS of 2.5 months for the Kd maintenance group (95% CI 0.47; 4.5, *p* = 0.016).

### Impact of missing data

Concerning informative drop-out, the pattern mixture models showed a statistically significant and clinically relevant difference for QLQ-C30-sum score reported by patients dropping out early compared to patients dropping out late for the Kd maintenance group (mean difference 12.2 points, 95% CI 5.3; 19.2, *p* = 0.001, with patients who dropped out early having poorer scores). There were no statistically significant or clinically relevant differences between early, late and never drop-out patients for the observation group or between never and late for the Kd maintenance group.

### Post hoc analysis

Our post hoc investigation revealed that 90% (2150 out of 2392) of the dexamethasone doses that were administered to the patients on protocol were full doses of 20 mg, and 83% (1983 out of 2392) of Kd maintenance doses were administered with full dose carfilzomib of 56 mg/sqm. In addition, 12% of the carfilzomib doses were administered as reduced doses (45, 36 or 27 mg/sqm), and for the remaining carfilzomib doses, treatment was omitted or data were not available. Further details of dose escalation and modification as well as administered doses of dexamethasone and carfilzomib can be found in the supplementary appendix Table S12, S13 and S14.

## Discussion

This is the first prospective randomized trial reporting patient-reported HRQL data in patients with relapsed MM receiving maintenance therapy with Kd after salvage ASCT. Kd maintenance therapy after salvage ASCT did not affect the perceptions of overall HRQL compared to observation, confirming our primary hypothesis. When adjusting the PFS for the HRQL impact, the Kd maintenance treatment still remained beneficial compared to observation, but reduced the PFS for the Kd maintenance group from 19.3 to 18.0 or 17.5 months and from 16.8 to 15.0 or 14.0 months for the observation group depending on the quality adjustment metric. Progression-free survival based on maintenance therapy should be quality-adjusted and constitutes a relevant estimate for the maintenance therapy benefits when taking the HRQL impact into account. For health technology assessment and health reimbursement decisions, the EORTC QLU-C10D score is suitable for estimating quality-adjusted PFS and for use in cost-utility analyses because it is a preference-based HRQL metric, whereas the EORTC QLQ-C30 summary score is not.

Patients randomized in the CARFI study were recovering after salvage ASCT. Evidence-based recovery trajectories in symptoms and functioning have been documented only following primary ASCT, but not for salvage ASCT [12–14]. Introducing maintenance therapy after ASCT leads to concerns of hampering patient recovery and/or initiating new side effects related to the drugs included in maintenance treatment. Our study showed that salvage ASCT recovery was hampered by Kd maintenance in six domains at eight months follow-up including physical functioning and fatigue, which are considered core HRQL domains in patients with multiple myeloma. Those findings were consolidated in our findings of higher proportions of patients treated with Kd maintenance reporting stable physical function at four months follow-up, which is a time point where improvement in physical functioning is expected, as well as worsening in social functioning at eight months follow-up.

Based on the non-infusion related toxicity profiles of carfilzomib and symptomatic side-effects of dexamethasone, we pre-specified domains and items expected to be affected during Kd maintenance treatment. The analysis showed that more patients reported insomnia, restlessness and agitation during Kd maintenance treatment. Those side-effects can primarily be attributed to dexamethasone treatment [45]. Our investigation of Kd-related symptoms revealed that dexamethasone symptomatic side-effects had a greater impact on patients than carfilzomib-related symptomatic side-effects. This finding in keeping with our post hoc analysis findings of 90% of dexamethasone doses being administered as full doses of 20 mg.

Dyspnoea is a well-documented side effect of carfilzomib, as revealed by the adverse events registrations from the large phase III clinical trials ASPIRE and ENDURANCE [39, 42]. Trajectories of patient-reported dyspnoea during carfilzomib treatment have to our knowledge not been published. Our investigation revealed no negative impact of carfilzomib maintenance on the patient-reported dyspnoea. As our findings of Kd-related symptoms are based on a patient cohort where the majority of patients received full-dose carfilzomib, dose reduction cannot be the exploration. However, in the CARFI study, the patients completed the HRQL questionnaires in the outpatient clinic on the drug administration day. The patients were asked to report symptoms experienced for the past seven days when completing the questionnaires. At that time point, it had been two weeks since the last dose of carfilzomib was administered, and the relevant side-effects might have subsided by then.

This study has several strengths. The objectives, endpoints, hypotheses and analyses methods are based on a pre-published statistical analysis plan guided by an evidence-based guideline for inclusion of PRO in clinical trials [31, 60]. This minimizes statistical multiplicity issues, avoids cherry picking of HRQL findings and ensures high-quality PRO data results to inform clinical decision-making. Another strength is the high PRO completion rate. These factors work together to confer scientific rigor and credibility to our results.

A limitation of related research rather than this study is that an MID for the primary PRO endpoint of the QLQ-C30-sum score is not yet established. An arbitrary cutoff of 10 points was thus chosen as the MID, based on previous EORTC QLQ-C30 MID findings in patients with MM [61]. However, looking at the domain results (where we did have MIDs [51]), we suspect that an MID for QLQ-C30-sum score lower than 10 points may have been more correct. Hence, this study might have been underpowered for detection of smaller, but potentially clinically important differences. The performed sensitivity analysis investigating the impact of missing data due to drop-out suggests that the missing data are not missing at random. The patients in the Kd maintenance group leaving the study late or who were on protocol at the end of the study period reported better HRQL compared to the patients leaving the protocol early. Reasons for drop-out were imbalanced between the two randomized groups during follow-up: 53 out of 86 (62%) patients in the observation group came off study protocol due to progressive disease, while the corresponding number was smaller in the Kd maintenance group (29 out of 82 (35%)). More patients in the Kd maintenance compared to the observation group (7 versus 1) left the study due to withdrawn consent.

## Conclusion

HRQL data from the phase II randomized CARFI trial demonstrate that Kd maintenance did not impair the patients´ perceptions of their overall HRQL, but Kd maintenance did delay recovery after salvage ASCT in several of the core domains of patients with multiple myeloma, specifically physical, role and social functioning, appetite loss, fatigue and body image. When adjusting the PFS for the HRQL impact, the Kd maintenance treatment still remained beneficial, and the difference in PFS between the two groups became larger when adjusting for HRQL. Progression-free survival of maintenance therapy should be quality-adjusted to be able to balance the benefits against the HRQL impact.

### Electronic supplementary material

Below is the link to the electronic supplementary material.

Supplementary Material 1

Supplementary Material 2

Supplementary Material 3

## Data Availability

The data used for this manuscript are available upon reasonable request directed to the corresponding author.
