# Supplementary material for: Health-related quality of life and quality-adjusted progression free survival for carfilzomib and dexamethasone maintenance following salvage autologous stem-cell transplantation in patients with multiple myeloma: a randomized phase 2 trial by the Nordic Myeloma Study Group
Source: J Patient Rep Outcomes. 2024 Feb 5;8:15. doi: 10.1186/s41687-024-00691-2 (PMC10844184; doi:10.1186/s41687-024-00691-2)
Supplement: Supplementary file 1 — Supplementary Material 1 [file 41687_2024_691_MOESM1_ESM.pdf]

CONSORT-Outcomes 2022 Extension items only (for separate completion of CONSORT 2010 and CONSORT-Outcomes 2022 items)

Section	Item No.	CONSORT-Outcomes item	Location Reported[b]
**Methods**			
Outcomes	6a.1	Provide a rationale for the selection of the domain for the trial's primary outcome	Manuscript page 7.
	6a.2	Describe the specific measurement variable (eg, systolic blood pressure), analysis metric (eg, change from baseline, final value, time to event), method of aggregation (eg, mean, proportion), and the time point for each outcome	Manuscript page 6 and 9-10.
	6a.3	If the analysis metric for the primary outcome represents within-subject change, define and justify the minimal important change in individuals	The primary outcomes is a between group analysis.
	6a.4	If the outcome data were continuous, but were analyzed as categorical (method of aggregation), specify the cutoff values used	Manuscript page 8-11.
	6a.5	If outcome assessments were performed at several time points after randomization, state the time points used for analysis	Manuscript page 5 and table 1.
	6a.6	If a composite outcome was used, define all individual components of the composite outcome	Manuscript page 9.
	6a.7	Identify any outcomes that were not prespecified in a trial registry or protocol	Manuscript page 5 and table 1.
	6a.8	Provide a description of the study instruments used to assess the outcome (eg, questionnaires, laboratory tests) along with reliability, validity, and responsiveness in a population similar to the study sample	Manuscript page 6-7 and table 2.
	6a.9	Describe who assessed the outcome (eg, nurse, parent), and any qualifications or trial-specific training necessary to administer the study instruments to assess the outcome	Manuscript page 7.
	6a.10	Describe any processes used to promote outcome data quality during data collection (eg, duplicate measurements) and after data collection (eg, range checks of outcome data values), or state where details can be found	Manuscript page 6-7.
Sample size	7a.1	Define and justify the target difference between treatment groups (eg, the minimal important difference)	Manuscript page 8-10.
Statistical methods	12a.1	Describe any methods used to account for multiplicity in the analysis or interpretation of the primary and secondary outcomes (eg, coprimary outcomes, same outcome assessed at multiple time points, or subgroup analyses of one outcome)	Manuscript page 5 and table 1, page 8-11.
	12a.2	State and justify any criteria for excluding any outcome data from the analysis and reporting, or report that no outcome data were excluded	No outcomes data were excluded.
	12a.3	Describe methods to assess patterns of missingness (eg, missing not at random), and describe the methods to handle missing outcome items or entire assessments	Manuscript page 10-11.

Section	Item No.	CONSORT-Outcomes item	Location Reported[b]
	12a.4	Provide definition of outcome analysis population relating to protocol nonadherence (eg, as randomized analysis)	Manuscript page 6 and 8-11.
**Results**			
Outcomes and estimation	17a.1	Include results for all prespecified outcome analyses or state where results can be found if not in this report	Manuscript results section page 11-14 includes all prespecified [illegible]
Ancillary analyses	18.1	If there were any analyses that were not prespecified, explain why they were performed	Manuscript page 14. Progression free survival [illegible]

[a]It is strongly recommended that this checklist be read in conjunction with the CONSORT-Outcomes and CONSORT Statement papers for important clarification on the items. The CONSORT Statement checklist is distributed under the terms of the Creative Commons Attribution License.
[b]Indicates page numbers and/or manuscript location: to be completed by authors during trial report development.
