# Supplementary material for: Health-related quality of life and quality-adjusted progression free survival for carfilzomib and dexamethasone maintenance following salvage autologous stem-cell transplantation in patients with multiple myeloma: a randomized phase 2 trial by the Nordic Myeloma Study Group
Source: J Patient Rep Outcomes. 2024 Feb 5;8:15. doi: 10.1186/s41687-024-00691-2 (PMC10844184; doi:10.1186/s41687-024-00691-2)
Supplement: Supplementary file 2 — Supplementary Material 2 [file 41687_2024_691_MOESM2_ESM.docx]

**Health-related quality of life and quality-adjusted progression free survival for carfilzomib and dexamethasone maintenance following salvage autologous stem-cell transplantation in patients with multiple myeloma:** A randomized phase 2 trial by the Nordic Myeloma Study Group

**ABSTRACT**

**Background** Decisions regarding maintenance therapy in patients with multiple myeloma should be based on both treatment efficacy and health-related quality of life (HRQL) consequences. In the CARFI trial, patients with first relapse of multiple myeloma underwent salvage autologous stem cell transplantation (salvage ASCT) before randomization to carfilzomib-dexamethasone maintenance therapy (Kd) or observation. The primary clinical endpoint was time to progression, which was extended by 8 months by Kd. The aim of this paper is to present the all HRQL endpoints of the CARFI trial including the HRQL effect of Kd maintenance therapy relative to observation. The primary HRQL endpoint was assessed by EORTC QLQ-C30 Summary score (QLQ-C30-sum) at 8 months follow-up. A key secondary HRQL endpoint was quality-adjusted progression-free-survival (QAPFS).

**Methods** HRQL was assessed with EORTC QLQ-C30, EORTC QLQ-MY20 and FACT/GOG-Ntx at randomization and every second month during follow-up. HRQL data were analyzed with linear mixed effect models until 8 months follow-up. QAPFS per individual was calculated by multiplying progression-free survival (PFS) by two quality-adjustment metrics, the QLQ-C30-sum and EORTC Quality of Life Utility Measure-Core 10 dimensions (QLU-C10D). The QAPFS per treatment group was estimated with the Kaplan-Meier method. P<0.05 was used for statistical significance, and a between-group minimal important difference of 10 points was interpreted as clinically relevant for the QLQ-C30-sum.

**Results** 168 patients were randomized. HRQL questionnaire compliance was 93%. For the QLQ-C30-sum, the difference of 4.62 points (95% confidence interval (CI) -8.9: -0.4, p=0.032) was not clinically relevant. PFS was 19.3 months for the Kd maintenance group and 16.8 months for the observation group; difference=2.5 months (95% CI 0.5; 4.5). QAPFS based on the QLQ-C30-sum for the Kd maintenance group was 18.0 months (95% CI 16.4; 19.6) and for the observation group 15.0 months (95% CI 13.5; 16.5); difference =3.0 months (95% CI 0.8–5.3). QAPFS based on the QLU-C10D for the Kd maintenance group was 17.5 months (95% CI 15.9; 19.2) and 14.0 months (95% CI 12.4; 15.5) for the observation group; difference =3.5 months (95% CI 1.1–5.9).

**Conclusions** Kd maintenance therapy after salvage ASCT did not adversely affect overall HRQL, but adjustment for HRQL reduced the PFS compared to unadjusted PFS. PFS of maintenance therapy should be quality-adjusted to balance the benefits and HRQL impact.

**BACKGROUND**

Primary endpoints in clinical cancer trials are preferably objective, well-defined and measurable outcomes, such as overall survival, progression free survival and response rates (1). However, secondary or exploratory endpoints, such as health-related quality of life (HRQL) measured through patient-reported outcomes (PROs), have become increasingly important to supplement the overall study findings (2). PROs provide a patient-focused assessment of the impact of a treatment on patients´ symptoms and functional abilities, which can inform regulatory label claims and clinical decision making (3-5).

Multiple myeloma (MM) is an incurable hematological cancer associated with bone destruction~~s~~, hypercalcemia, anemia, renal failure and infections (6). Overall survival has markedly increased after the introduction of novel treatments in the past two decades (7-9). A contributor to improved overall survival is high-dose melphalan with autologous stem-cell transplantation (ASCT), which is standard treatment in patients with newly diagnosed MM younger than 70 years of age without significant comorbidities (10, 11). High-dose melphalan causes acute toxicity of anorexia, mucositis with pain and diarrhea, neutropenia and thrombocytopenia with temporary impairment of HRQL and full recovery for some patients, 1-2 months post-ASCT, while other patients still have moderate to severe symptoms one year post-ASCT (12-15).

Another contributor to improved overall survival in MM is maintenance treatment, i.e. long term therapy that extends response duration (16-18). An important consideration for individual decision-making about maintenance therapy is the long-term impact on patient HRQL (17, 19). Results from secondary PRO endpoints have shown that HRQL during maintenance varies with the specific drug used. Despite thalidomide´s prolongation of progression free survival (PFS), this drug is not approved for maintenance therapy for MM due to unacceptable impairment of HRQL (20, 21). Lenalidomide maintenance improves PFS and overall survival and is approved for maintenance therapy in transplant-eligible patients after ASCT (18). Adverse event registration from clinical trials shows that lenalidomide maintenance after ASCT is tolerable, and includes mainly hematological side-effects, diarrhea and fatigue (22-24). However, randomized studies comparing HRQL during lenalidomide maintenance versus observation after ASCT are lacking. Published studies allowed several drugs as maintenance therapy or were designed with another drug as comparator (25-27). In one of the studies, HRQL comparison between patients receiving maintenance therapy versus no maintenance therapy after first-line ASCT was done (27). Several maintenance drugs were included, but not carfilzomib. The overall results indicated minimal impact on HRQL by maintenance therapy, but with worsening diarrhea and reduction in future perspectives.

Eventually, almost all patients with MM will experience relapse or progressive disease and for these patients, salvage ASCT will be an option, particularly for patients who achieved a long remission after frontline ASCT (10, 28). After successful salvage ASCT, it would be desirable to prolong response duration with maintenance therapy, as long as that does not compromise HRQL or hamper recovery after salvage ASCT. However, there is limited randomized evidence on maintenance therapy after salvage ASCT. In the ReLApsE trial, lenalidomide maintenance therapy was included in the transplant arm; but the study was designed to evaluate salvage ASCT, not maintenance therapy (29).

To investigate the impact of maintenance therapy after salvage ASCT, the Nordic Myeloma Study Group initiated the CARFI trial in 2015 (30). Carfilzomib, a second-generation proteasome inhibitor, was chosen for maintenance therapy in the CARFI trial, administered with dexamethasone every second week. The primary clinical endpoint of the CARFI study was time to progression; as previously reported, time to progression was significantly prolonged by eight months for the carfilzomib-dexamethasone (Kd) maintenance group (25.1 months) compared to observation (16.7 months) (30). HRQL was one of the secondary endpoints of the CARFI trial; results of the European Organisation for Research and Treatment of Cancer (EORTC) Quality of Life Questionnaire – Core 30 items (QLQ-C30) global health status/quality of life (GHS/QoL) subscale showed no between-group difference during the 2-year follow-up period (30). The aim of this paper is to present an in-depth analysis of the CARFI trial PRO endpoints, as per the statistical analysis plan (31).

**PRO objective, endpoints and hypotheses**

The objective of the HRQL component of the CARFI trial was to assess the impact of Kd maintenance therapy, relative to observation, post salvage ASCT on a range of relevant aspects of HRQL. The primary PRO endpoint was change in overall HRQL from randomization to eight months follow-up, with the corresponding primary PRO hypothesis that there was no difference between the Kd maintenance therapy and observation groups. The primary PRO endpoint was augmented by the average overall HRQL per-patient based on all completed HRQL forms from randomization to last follow-up time-point prior to progressive disease/death/drug discontinuation/end of study (whichever came first). These two primary endpoints were supplemented by secondary PRO endpoints defined in terms of specific HRQL domains from randomization to 8 months later: 1) mean change; 2) the proportion of patients who improved, remained stable or worsened; 3) time to first recorded improvement; and 4) the proportion of patients with Kd-related symptoms. The final secondary endpoint was quality-adjusted progression free survival (QAPFS) from randomization to progressive disease/death/drug discontinuation/end of study (whichever came first). Hypotheses corresponding to each of these endpoints, including specific HRQL domains and symptoms, the questionnaires used to assess these, and the analysis and interpretation approach are presented in Table 1.

**METHODS**

**Design, patients and treatment**

The CARFI trial was a Nordic Myeloma Study Group open-label multi-center, randomized, phase II clinical trial, using a parallel (1:1) group design (clinicaltrials.gov: NCT02572492). Details of the study design, trial treatment and main findings have been published elsewhere (30). In brief, patients with MM aged 18 years or more with first relapse after prior ASCT and found eligible for salvage ASCT were included at 25 hospitals within four Nordic countries and Lithuania from January 2015 to April 2018. Key exclusion criteria were previous treatment with carfilzomib, maintenance treatment given after first ASCT, World Health Organization performance status ≥3, significant neuropathy (grade 3-4, or grade 2 with pain) and any comorbidity that would preclude treatment with carfilzomib or salvage ASCT.

Two months after reinduction with four cycles of carfilzomib-lenalidomide-dexamethasone followed by salvage ASCT, patients were randomized to Kd maintenance or observation. Kd maintenance was given as intravenous carfilzomib (27 mg/sqm every second week with escalation of carfilzomib to 56 mg/sqm if tolerated) and oral dexamethasone 20 mg every second week until progression, unacceptable adverse effects, withdrawal of consent or 1 September 2019 (end of study). Dose modifications of carfilzomib and/or dexamethasone were done in case of toxicity related to the drugs (described in the supplementary appendix).

**PRO instruments**

HRQL were assessed with three instruments: the EORTC core module (QLQ-C30) (32, 33) and Multiple Myeloma module (QLQ-MY20) (34), and the Functional Assessment of cancer Therapy/Gynaecologic Oncolocy Group-Neurotoxity (FACT/GOG-ntx) instruments (35). The three questionnaires and domains are described in Table 2.

**PRO data collection**

Patient inclusion in the PRO sub-protocol was part of the CARFI trial. HRQL was assessed with all three instruments at randomization (two months post salvage ASCT) and then every second month in the outpatient clinic at study visits. The study nurses had access to trial specific guidance in PRO data collection. If a scheduled visit was postponed (e.g. due to vacation, public holidays, acute toxicities beyond grade 2), the PRO data collection was postponed as well. From study initiation until July 2017, the study nurses administered all three PRO instruments on paper for the patients to complete in the clinic. From July 2017, electronic completion of the EORTC instruments via a tablet directly into a REDCap database became an option. Real-time monitoring of non-completion of instruments was not carried out. PRO data collection continued until end of study for each patient, defined as the time of disease progression, death, drug discontinuation or study termination (whichever came first). Study termination was defined as the last protocol visit for the last included patient, which was on 1 September 2019.

**Rationale for PRO endpoints**

In developing the statistical analysis plan, HRQL studies of patients with MM were reviewed to inform our choice of PRO endpoints and appropriate PRO measures (31). The EORTC QLQ-C30 Summary (QLQ-C30-sum) score was chosen to assess the primary PRO endpoint because there is no standard HRQL assessment for use in MM maintenance trials, it captures the generic HRQL concepts of importance in cancer, it reduces issues with multiplicity (type I error), and it has been validated in patients with hematological malignancies (36, 37). Secondary PRO endpoints were specified a priori, aligned with hypotheses (Table 1), assessed by specific domains from the QLQ-C30, QLQ-MY20 and FACT/GOG-ntx. These included four of the functioning domains (physical, role, social, emotional) and GHS/QoL from EORTC QLQ-C30, and two domains from the QLQ-MY20 (body image and future perspectives); these were chosen because they have previously been shown to be affected after primary ASCT (12-14). Based on the literature, Kd-related symptoms were identified and defined as fatigue, dyspnea, nausea/vomiting, diarrhea, insomnia, agitation and restlessness; these were included to assess the non-infusion related symptomatic impact of carfilzomib and dexamethasone treatment (38-45). Time in QAPFS was included as it captures the benefits and consequences of Kd maintenance therapy in terms of both treatment efficacy (PFS) and HRQL consequences. Further details are provided in the statistical analysis plan (31).

**Statistical analyses**

The statistical analysis plan for the PRO endpoints was finalized and published before starting the PRO data analyses (31); a clarifying amendment was added to the published statistical analysis plan before finalizing the PRO data analyses. The statistical analyses are described here in brief. For each patient, the study period was from randomization to that patient’s last follow-up prior to progressive disease/death/drug discontinuation/end of study (which ever came first). The analyses were based on all available questionnaires from randomized patients on protocol until last follow-up time point except for the supportive analysis of the primary endpoint where all available questionnaires were included in the analysis. The last follow-up time point was defined as the time point *before* the number of patients on protocol in one of the groups became less than 15 patients. Analyses were carried out using Stata 17.

Patient characteristics and PRO scores at randomization were described with summary statistics (mean and 95% confidence intervals (CI)). PRO completion rates were calculated for each assessment time point as number of randomized patients with enough completed items to calculate the QLQ-C30-sum score as a proportion of patients on protocol at that time point.

HRQL domain scores of the QLQ-C30 and QLQ-MY20 were calculated according to EORTC standard scoring (46). The QLQ-C30-sum score was calculated from the mean of 13 of the 15 QLQ-C30 domains (excluding GHS/QoL and Financial impact defining items) (37). The QLQ-C30-sum score ranges from 0 to 100, with 100 being best. The FACT/GOG-ntx score was calculated according to the FACT manual (47).

For the endpoints involving change in HRQL, the longitudinal PRO data were analyzed with a linear mixed effect model for repeated measures. The model included patients as a random variable and time (baseline, two, four, six and eight months), treatment group and country as fixed factors. The results were tested for statistical significance at the 5% level for the primary HRQL endpoint and at the 1% level for the secondary HRQL endpoints. Statistically significant results were interpreted as clinically relevant/meaningful if they exceed the threshold for minimally important difference/change (MID). As MID has not yet been established for the QLQ-C30-sum score, a 10-point change/difference was pre-specified as indicating a clinically relevant change (within a group) and a clinically relevant difference in change between groups (48, 49). Within~~-~~ and between group changes in the individual domains of the QLQ-C30 questionnaire were interpreted according to the evidence-based guidelines for between-group differences (50) and change over time (51) using the threshold for a small difference/change. For the domains of QLQ-MY20, recommended estimates for MIDs were used (disease symptoms and side effects of treatment; 10 points, body image; 13 points and future perspectives: 9 points) (52). For the FACT/GOG-ntx subscale, 4.4 (53) points were used as primary MID and 11.8 points were used for sensitivity analysis (54).

For the supportive analysis of the primary endpoint, the average per-patient QLQ-C30-sum score from randomization to progressive disease/death/drug discontinuation/end of study was calculated. The mean per-patient QLQ-C30-sum score was calculated for each group and compared with a rank-sum test.

To evaluate the proportion of patients who improved, remained stable or worsened in HRQL, patients were categorized as having perceived either an improvement or worsening from randomization if they experienced a change in score (in the direction of improvement or worsening, respectively) that exceeded at least 20 points (55). For the single item domain of body image, 33 points were used (52).

Time to first recorded improvement in HRQL was calculated for each patient as time from randomization to the first time the patient reported an improvement of at least 20 points for the EORTC multi-item domains (55), and 33 points for the body image single item domain (52). Patients who did not record an improvement at any time point were censored at end of study. Patients with no score at randomization and patients with high functioning as well as good GHS/QoL (greater than 80 points) and body image (less than 33 points) at randomization were excluded from this analysis since this left no room for improvement. The mean time to improvement was compared between groups using a proportional hazards Cox regression model with treatment, PRO score at randomization and country as covariates, and presented as a hazard ratio (HR) with corresponding 95% CI. A post-hoc analysis of average time of first recorded improvement with corresponding standard deviations was performed.

For endpoints involving the proportion of patients with Kd-related symptoms, raw item scores were converted to 0-100 scales according to the EORTC manual (46), i.e. “none at all”=0, “a little”=33, “quite a bit”=67, “very much”=100. The proportion of patients with Kd-related symptoms was defined as the number of patients experiencing any of the Kd-related symptoms corresponding to at least “a little” (≥33 points) divided by the total number of patients. Similarly for the proportion of patients reporting moderate to severe Kd-related symptoms, a score of at least “quite a bit” (≥66 points) was used. This dichotomized grading has previously been used in patients with hematological malignancies (56). Differences between treatment groups in these proportions were analyzed as binary endpoints using chi-square tests of homogeneity.

Preference-based utility scores were calculated from the QLQ-C30 data using the utility scoring algorithm of EORTC Quality of Life Utility Measure-Core 10 dimensions (QLU-C10D) (57), which yields a score with a maximum of one (representing the best possible health state), and is anchored at zero (representing the state of being dead). Implementing this algorithm requires a country-specific value set; as a Danish QLU-C10D value set was not available at the time of analysis, the German value set was used (58), as it was considered to be closest culturally of the country-specific value sets available at the time of analysis. The time in QAPFS was calculated using two quality adjustment metrics: the per-patient mean EORTC QLQ-C30-sum score (divided by 100, as required for quality adjustment of life years) and the per-patient mean QLU-C10D utility score (59). Patient-level QAPFS was calculated by multiplying the per-patient estimated mean time to progression/death (censored at last follow-up time point) by each of the quality adjustment metrics. Group-level QAPFS was then estimated with the Kaplan-Meier method. The mean difference in QAPFS between the two groups was estimated using bootstrap methods for each quality-adjustment metric.

To examine potentially missing data patterns (informative drop-out) two pattern mixture model analyses were performed; 1) stratifying patients into two groups based on drop-out time early (drop-out between zero and eight months follow-up) and late (participated in the study at 10 months) per treatment group, 2) stratifying patients into three groups based on drop-out time (early, late and never drop-out) per treatment group.

A post-hoc analysis was performed to investigate compliance with Kd maintenance therapy by assessing the proportion of fully administered, reduced and omitted doses of carfilzomib and dexamethasone relative to scheduled doses.

Sample size in the maintenance phase of the CARFI trial was determined according to the primary trial endpoint (time to progression). According to the trial protocol, it was expected that 150 patients would continue to the maintenance phase. No formal power analysis was performed for the PROs, and no patients were involved in the design or the interpretation phase of the study.

**RESULTS**

**Patient population**

In total, 181 patients underwent salvage ASCT, and 168 patients continued to randomization between Kd maintenance or observation; 82 were randomized to Kd maintenance and 86 to observation, constituting the population for this HRQL analysis. Reasons for drop-out before randomization have been reported previously (30). Reasons for drop-out from randomization until eight months follow-up are presented in the CONSORT flow diagram of Figure 1 and in the supplementary appendix Figure S1 until last follow-up time point (i.e. 22 months). Patient demographics, clinical characteristics and PRO mean scores at randomization are presented in Table 3. PRO completion rate until eight months follow-up was 93% (95% for the Kd maintenance and 91% for the observation group) and 93% until last follow-up time point (supplementary appendix Table S1).

**Effect of Kd maintenance on HRQL**

The difference in mean change from randomization between groups in QLQ-C30-sum score estimated at eight months was 4.62 points on the 0-100 scale (95% CI -8.9; -0.4, p=0.032) was statistically significant but not clinically important (i.e. it was less than the predefined MID). The findings are visualized in Figure 2. Similarly, none of the differences at specific time points (two, four and six months follow-up) reached statistical significance and clinical relevance. Data until last follow-up time point are provided in the supplemental appendix Table S2. Results of the pre-specified supportive analysis of the primary endpoint, the average per-patient mean QLQ-C30-sum score, was 81.4 (SD 12.9) for the Kd maintenance group and 82.5 (SD 12.3) for the observation group. These means were similar and did not differ statistically significantly between the groups (rank sum test, p=0.625).

**Effect of Kd maintenance on the individual PRO domains**

Within the observation group, the patients reported statistically significant and clinically meaningful improvements at eight months in six domains (physical, role, social functioning, appetite loss, fatigue and body image), whereas the patients in the Kd maintenance group did not report statistically significant or clinically meaningful improvements in any domains at eight months follow-up. The findings are presented in Table 4. For these six domains, the results at eight months are presented in Figure 3, and mean score changes, 95% confidence intervals and p-values until last follow-up time point are presented in supplementary appendix Table S3-S8.

**Proportion of patients who improved, remained stable or worsened**

Generally, the majority (range 52%-93%) of patients in both groups remained stable from randomization to the eight months follow-up, with some notable exceptions. A higher proportion of patients in the Kd maintenance compared to the observation group (93% versus 81%) reported stable physical functioning at four months (odds ratio 2.99, 95% CI 1.01; 8.83, p=0.040). Also, a higher proportion of patients in the Kd maintenance group compared to the observation group (13% versus 2%) reported worsening in social functioning at eight months (odds ratio 8.74, 95% CI 1.06; 72.2, p=0.018). The proportions and odds ratios for all domains from randomization to two, four, six and eight months follow-up are reported in Supplementary Table S9.

**Time to first recorded improvement**

There were no statistically significant between-group difference in time to first recorded improvement in any of the (EORTC QLQ-C30) functional domains, GHS/QoL scale, QLQ-MY20 body image domain or future perspectives (Table 5). The average weeks to first recorded improvement is displayed in the supplementary appendix Table S10.

**Proportion of patients with Kd-related symptoms**

Significantly more patients in the Kd maintenance group developed Kd-related symptoms of restlessness and agitation (odds ratio 1.90, CI95% 1.01; 3.58, p=0.046) and insomnia (odds ratio 2.60, CI 95% 1.15; 5.88, p=0.019) during the study period (Figure 4 and the supplementary appendix Table S11).

**Quality-adjusted progression free survival**

When quality-adjusting the PFS based on the QLQ-C30-sum score, the QAPFS difference between the two groups was 3.0 months in favor of the Kd maintenance group (95% CI 0.78; 5.28, p=0.008). Mean QAPFS based on the QLQ-C30-sum score for the Kd maintenance group was 18.0 months (95% CI 16.4; 19.6) and 15.0 months for the observation group (95% CI 13.5; 16.5). Similarly for QAPFS based on the QLU-C10D, where Kd maintenance significantly extended QAPFS by 3.5 months (95% CI 1.2; 5.9, p=0.004) compared to observation. The mean QAPFS based on the QLU-C10D for patients in the Kd maintenance group was 17.5 months (95% CI 15.9; 19.2) and 14.0 months (95% CI 12.4; 15.5) for the observation group. The quality-adjusted progression-free-survival curves are displayed in Figure 5. For comparison, the unadjusted progression-free survival was 19.3 months for the Kd maintenance group (95% CI 17.9; 20.7) and 16.8 for the observation group (95% CI 15.4; 18.3) resulting in a significantly superior PFS of 2.5 months for the Kd maintenance group (95% CI 0.47; 4.5, p=0.016).

**Impact of missing data**

Concerning informative drop-out, the pattern mixture models showed a statistically significant and clinically relevant difference for QLQ-C30-sum score reported by patients dropping out early compared to patients dropping out late for the Kd maintenance group (mean difference 12.2 points, 95% CI 5.3; 19.2, p=0.001, with patients who dropped out early having poorer scores). There were no statistically significant or clinically relevant differences between early, late and never drop-out patients for the observation group or between never and late for the Kd maintenance group.

**Post hoc analysis**

Our post hoc investigation revealed that 90% (2150 out of 2392) of the dexamethasone doses that were administered to the patients on protocol were full doses of 20 mg, and 83% (1983 out of 2392) of Kd maintenance doses were administered with full dose carfilzomib of 56 mg/sqm. In addition, 12% of the carfilzomib doses were administered as reduced doses (45, 36 or 27 mg/sqm), and for the remaining carfilzomib doses, treatment was omitted or data were not available. Further details of dose escalation and modification as well as administered doses of dexamethasone and carfilzomib can be found in the supplementary appendix Table S12, S13 and S14.

**DISCUSSION**

This is the first prospective randomized trial reporting patient-reported HRQL data in patients with relapsed MM ~~multiple myeloma~~ receiving maintenance therapy with Kd after salvage ASCT. Kd maintenance therapy after salvage ASCT did not affect the perceptions of overall HRQL compared to observation, confirming our primary hypothesis. When adjusting the PFS for the HRQL impact, the Kd maintenance treatment still remained beneficial compared to observation, but reduced the PFS for the Kd maintenance group from 19.3 to 18.0 or 17.5 months and from 16.8 to 15.0 or 14.0 months for the observation group depending on the quality adjustment metric. Progression-free survival based on maintenance therapy should be quality-adjusted and constitutes a relevant estimate for the maintenance therapy benefits when taking the HRQL impact into account. For health technology assessment and health reimbursement decisions, the EORTC QLU-C10D score is suitable for estimating quality-adjusted PFS and for use in cost-utility analyses because it is a preference-based HRQL metric, whereas the EORTC QLQ-C30 summary score is not.

Patients randomized in the CARFI study were recovering after salvage ASCT. Evidence-based recovery trajectories in symptoms and functioning have been documented only following primary ASCT, but not for salvage ASCT (12-14). Introducing maintenance therapy after ASCT leads to concerns of hampering patient recovery and/or initiating new side effects related to the drugs included in maintenance treatment. Our study showed that salvage ASCT recovery was hampered by Kd maintenance in six domains at eight months follow-up including physical functioning and fatigue, which are considered core HRQL domains in patients with multiple myeloma. Those findings were consolidated in our findings of higher proportions of patients treated with Kd maintenance reporting stable physical function at four months follow-up, which is a time point where improvement in physical functioning is expected, as well as worsening in social functioning at eight months follow-up.

Based on the non-infusion related toxicity profiles of carfilzomib and symptomatic side-effects of dexamethasone, we pre-specified domains and items expected to be affected during Kd maintenance treatment. The analysis showed that more patients reported insomnia, restlessness and agitation during Kd maintenance treatment. Those side-effects can primarily be attributed to dexamethasone treatment (45). Our investigation of Kd-related symptoms revealed that dexamethasone symptomatic side-effects had a greater impact on patients than carfilzomib-related symptomatic side-effects. This finding in keeping with our post hoc analysis findings of 90% of dexamethasone doses being administered as full doses of 20 mg.

Dyspnoea is a well-documented side effect of carfilzomib, as revealed by the adverse events registrations from the large phase III clinical trials ASPIRE and ENDURANCE (39, 42). Trajectories of patient-reported dyspnoea during carfilzomib treatment have to our knowledge not been published. Our investigation revealed no negative impact of carfilzomib maintenance on the patient-reported dyspnoea. As our findings of Kd-related symptoms are based on a patient cohort where the majority of patients received full-dose carfilzomib, dose reduction cannot be the exploration. However, in the CARFI study, the patients completed the HRQL questionnaires in the outpatient clinic on the drug administration day. The patients were asked to report symptoms experienced for the past seven days when completing the questionnaires. At that time point, it had been two weeks since the last dose of carfilzomib was administered, and the relevant side-effects might have subsided by then.

This study has several strengths. The objectives, endpoints, hypotheses and analyses methods are based on a pre-published statistical analysis plan guided by an evidence-based guideline for inclusion of PRO in clinical trials (31, 60). This minimizes statistical multiplicity issues, avoids cherry picking of HRQL findings and ensures high-quality PRO data results to inform clinical decision-making. Another strength is the high PRO completion rate. These factors work together to confer scientific rigor and credibility to our results.

A limitation of related research rather than this study is that an MID for the primary PRO endpoint of the QLQ-C30-sum score is not yet established. An arbitrary cutoff of 10 points was thus chosen as the MID, based on previous EORTC QLQ-C30 MID findings in patients with MM (61). However, looking at the domain results (where we did have MIDs (51)), we suspect that an MID for QLQ-C30-sum score lower than 10 points may have been more correct. Hence, this study might have been underpowered for detection of smaller, but potentially clinically important differences. The performed sensitivity analysis investigating the impact of missing data due to drop-out suggests that the missing data are not missing at random. The patients in the Kd maintenance group leaving the study late or who were on protocol at the end of the study period reported better HRQL compared to the patients leaving the protocol early. Reasons for drop-out were imbalanced between the two randomized groups during follow-up: 53 out of 86 (62%) patients in the observation group came off study protocol due to progressive disease, while the corresponding number was smaller in the Kd maintenance group (29 out of 82 (35%)). More patients in the Kd maintenance compared to the observation group (7 versus 1) left the study due to withdrawn consent.

**Conclusion**

HRQL data from the phase II randomized CARFI trial demonstrate that Kd maintenance did not impair the patients´ perceptions of their overall HRQL, but Kd maintenance did delay recovery after salvage ASCT in several of the core domains of patients with multiple myeloma, specifically physical, role and social functioning, appetite loss, fatigue and body image. When adjusting the PFS for the HRQL impact, the Kd maintenance treatment still remained beneficial, and the difference in PFS between the two groups became larger when adjusting for HRQL. Progression-free survival of maintenance therapy should be quality-adjusted to be able to balance the benefits against the HRQL impact.

**List of abbreviations**

**ASCT** autologous stem cell transplantation

**QLQ-C30** Quality of Life Questionnaire Core 30 items

**QLQ-MY20** Multiple Myeloma Module

**CI** confidence interval

**EORTC** European Organisation for Research and Treatment of Cancer

**FACT/GOG-Ntx** Functional Assessment of Cancer Therapy/Gynaecologic Oncology Group/Neurotoxicity

**GHS/QoL** Global health status/Quality of life

**HRQL** health-related quality of life

**Kd** carfilzomib-dexamethasone

**MID** minimally important difference

**MM** multiple myeloma

**MMRM** mixed effects model for repeated measures

**PFS** progression free survival

**PRO** patient-reported outcome

**QAPFS** quality-adjusted progression free survival

**QoL** quality of life

**QLU-C10D** Quality of Life Utility Measure-Core 10 dimensions

**QLQ-C30-sum** QLQ-C30 Summary score

**References**

1. Delgado A, Guddati AK. Clinical endpoints in oncology - a primer. Am J Cancer Res. 2021;11(4):1121-31.

2. Bottomley A, Reijneveld JC, Koller M, Flechtner H, Tomaszewski KA, Greimel E. Current state of quality of life and patient-reported outcomes research. Eur J Cancer. 2019;121:55-63.

3. US Department of Health and Human Services Food and Drug Administration. US Food and Drug Administration. Guidance for industry: patient-reported outcome measures: Use in medical product development to support labeling claims. <https://www.fda.gov/downloads/Drugs/GuidanceComplianceRegulatoryInformation/Guidances/UCM193282.pdf> [updated December 2009.

4. European Medicine Agency. The use of patientreported outcome (PRO) measures in oncology studies. Appendix 2 to the guideline on the evaluation of anticancer medicinal products in man. <http://www.ema.europa.eu/docs/en_GB/document_library/Other/2016/04/WC500205159.pdf2016> [

5. Cella D, Chen CI, Quek RGW, Uribarren A, Reaney M, Mastey V, et al. Patient-reported outcomes labeling for oncology drugs: Multidisciplinary perspectives on current status and future directions. Front Pharmacol. 2022;13:1031992.

6. Palumbo A, Anderson K. Multiple myeloma. N Engl J Med. 2011;364(11):1046-60.

7. Langseth Ø O, Myklebust T, Johannesen TB, Hjertner Ø, Waage A. Incidence and survival of multiple myeloma: a population-based study of 10 524 patients diagnosed 1982-2017. Br J Haematol. 2020;191(3):418-25.

8. Hannig LH, Nielsen LK, Ibsen R, Arnheim-Dahlström L, Kjellberg J, Abildgaard N. The impact of changed treatment patterns in multiple myeloma on health-care utilisation and costs, myeloma complications, and survival: A population-based comparison between two time periods in Denmark. Eur J Haematol. 2021;107(1):63-73.

9. Thorsteinsdottir S, Dickman PW, Landgren O, Blimark C, Hultcrantz M, Turesson I, et al. Dramatically improved survival in multiple myeloma patients in the recent decade: results from a Swedish population-based study. Haematologica. 2018;103(9):e412-e5.

10. Giralt S, Garderet L, Durie B, Cook G, Gahrton G, Bruno B, et al. American Society of Blood and Marrow Transplantation, European Society of Blood and Marrow Transplantation, Blood and Marrow Transplant Clinical Trials Network, and International Myeloma Working Group Consensus Conference on Salvage Hematopoietic Cell Transplantation in Patients with Relapsed Multiple Myeloma. Biol Blood Marrow Transplant. 2015;21(12):2039-51.

11. Lenhoff S, Hjorth M, Holmberg E, Turesson I, Westin J, Nielsen JL, et al. Impact on survival of high-dose therapy with autologous stem cell support in patients younger than 60 years with newly diagnosed multiple myeloma: a population-based study. Nordic Myeloma Study Group. Blood. 2000;95(1):7-11.

12. Chakraborty R, Hamilton BK, Hashmi SK, Kumar SK, Majhail NS. Health-Related Quality of Life after Autologous Stem Cell Transplantation for Multiple Myeloma. Biol Blood Marrow Transplant. 2018.

13. D'Souza A, Brazauskas R, Stadtmauer EA, Pasquini MC, Hari P, Bashey A, et al. Trajectories of quality of life recovery and symptom burden after autologous hematopoietic cell transplantation in multiple myeloma. Am J Hematol. 2023;98(1):140-7.

14. Ebraheem MS, Seow H, Balitsky AK, Pond GR, Wildes TM, Sivapathasundaram B, et al. Trajectory of Symptoms in Patients Undergoing Autologous Stem Cell Transplant for Multiple Myeloma: A Population-Based Cohort Study of Patient-Reported Outcomes. Clin Lymphoma Myeloma Leuk. 2021;21(9):e714-e21.

15. Cunningham D, Paz-Ares L, Gore ME, Malpas J, Hickish T, Nicolson M, et al. High-dose melphalan for multiple myeloma: long-term follow-up data. J Clin Oncol. 1994;12(4):764-8.

16. Zhi Y, Bao S, Mao J, Chai G, Zhu J, Liu C, et al. Efficacy of maintenance treatment in patients with multiple myeloma: a systematic review and network meta-analysis. Hematology (Amsterdam, Netherlands). 2022;27(1):1069-88.

17. Karam D, Gertz M, Lacy M, Dispenzieri A, Hayman S, Dingli D, et al. Impact of maintenance therapy post autologous stem cell transplantation for multiple myeloma in early and delayed transplant. Bone Marrow Transplant. 2022;57(5):803-9.

18. McCarthy PL, Holstein SA, Petrucci MT, Richardson PG, Hulin C, Tosi P, et al. Lenalidomide Maintenance After Autologous Stem-Cell Transplantation in Newly Diagnosed Multiple Myeloma: A Meta-Analysis. Journal of clinical oncology. 2017;35(29):3279-89.

19. Ludwig H, Durie BG, McCarthy P, Palumbo A, San Miguel J, Barlogie B, et al. IMWG consensus on maintenance therapy in multiple myeloma. Blood. 2012;119(13):3003-15.

20. Stewart AK, Trudel S, Bahlis NJ, White D, Sabry W, Belch A, et al. A randomized phase 3 trial of thalidomide and prednisone as maintenance therapy after ASCT in patients with MM with a quality-of-life assessment: the National Cancer Institute of Canada Clinicals Trials Group Myeloma 10 Trial. Blood. 2013;121(9):1517-23.

21. Nielsen LK, Stege C, Lissenberg-Witte B, van der Holt B, Mellqvist UH, Salomo M, et al. Health-related quality of life in transplant ineligible newly diagnosed multiple myeloma patients treated with either thalidomide or lenalidomide-based regimen until progression: a prospective, open-label, multicenter, randomized, phase 3 study. Haematologica. 2020;105(6):1650-9.

22. McCarthy PL, Owzar K, Hofmeister CC, Hurd DD, Hassoun H, Richardson PG, et al. Lenalidomide after stem-cell transplantation for multiple myeloma. N Engl J Med. 2012;366(19):1770-81.

23. Attal M, Lauwers-Cances V, Marit G, Caillot D, Moreau P, Facon T, et al. Lenalidomide maintenance after stem-cell transplantation for multiple myeloma. N Engl J Med. 2012;366(19):1782-91.

24. Palumbo A, Cavallo F, Gay F, Di Raimondo F, Ben Yehuda D, Petrucci MT, et al. Autologous transplantation and maintenance therapy in multiple myeloma. N Engl J Med. 2014;371(10):895-905.

25. Abonour R, Wagner L, Durie BGM, Jagannath S, Narang M, Terebelo HR, et al. Impact of post-transplantation maintenance therapy on health-related quality of life in patients with multiple myeloma: data from the Connect(R) MM Registry. Ann Hematol. 2018.

26. Roussel M, Hebraud B, Hulin C, Perrot A, Caillot D, Stoppa AM, et al. Health-related quality of life results from the IFM 2009 trial: treatment with lenalidomide, bortezomib, and dexamethasone in transplant-eligible patients with newly diagnosed multiple myeloma. Leuk Lymphoma. 2020;61(6):1323-33.

27. Tay J, Vij R, Norkin M, Buadi F, Kindwall-Keller TL, Roberts JS, et al. Health related quality of life for multiple myeloma patients according to treatment strategy after autologous stem cell transplant: a cross-sectional study using EORTC, EQ-5D and MY-20 scales. (1029-2403 (Electronic)).

28. Gimsing P, Hjertner Ø, Abildgaard N, Andersen NF, Dahl TG, Gregersen H, et al. Salvage bortezomib-dexamethasone and high-dose melphalan (HDM) and autologous stem cell support (ASCT) in myeloma patients at first relapse after HDM with ASCT. A phase-2 trial. Bone Marrow Transplant. 2015;50(10):1306-11.

29. Goldschmidt H, Baertsch MA, Schlenzka J, Becker N, Habermehl C, Hielscher T, et al. Salvage autologous transplant and lenalidomide maintenance vs. lenalidomide/dexamethasone for relapsed multiple myeloma: the randomized GMMG phase III trial ReLApsE. Leukemia. 2021;35(4):1134-44.

30. Gregersen H, Peceliunas V, Remes K, Schjesvold F, Abildgaard N, Nahi H, et al. Carfilzomib and dexamethasone maintenance following salvage ASCT in multiple myeloma: A randomised phase 2 trial by the Nordic Myeloma Study Group. Eur J Haematol. 2022;108(1):34-44.

31. Eshoj HR, King M, Abildgaard N, Gregersen H, Nielsen LK, Möller S. Statistical Analysis Plan (SAP) for the CARFI trial - Analysis of secondary endpoints: patient-reported outcomes <https://portal.findresearcher.sdu.dk/da/publications/statistical-analysis-plan-sap-for-the-carfi-trial-analysis-of-sec> 2020

32. Aaronson NK, Ahmedzai S, Bergman B, Bullinger M, Cull A, Duez NJ, et al. The European Organization for Research and Treatment of Cancer QLQ-C30: a quality-of-life instrument for use in international clinical trials in oncology. Journal of the National Cancer Institute. 1993;85(5):365-76.

33. Wisloff F, Eika S, Hippe E, Hjorth M, Holmberg E, Kaasa S, et al. Measurement of health-related quality of life in multiple myeloma. Nordic Myeloma Study Group. British journal of haematology. 1996;92(3):604-13.

34. Cocks K, Cohen D, Wisløff F, Sezer O, Lee S, Hippe E, et al. An international field study of the reliability and validity of a disease-specific questionnaire module (the QLQ-MY20) in assessing the quality of life of patients with multiple myeloma. Eur J Cancer. 2007;43(11):1670-8.

35. Huang HQ, Brady MF, Cella D, Fleming G. Validation and reduction of FACT/GOG-Ntx subscale for platinum/paclitaxel-induced neurologic symptoms: a gynecologic oncology group study. International journal of gynecological cancer : official journal of the International Gynecological Cancer Society. 2007;17(2):387-93.

36. Efficace F, Cottone F, Sommer K, Kieffer J, Aaronson N, Fayers P, et al. Validation of the European Organisation for Research and Treatment of Cancer Quality of Life Questionnaire Core 30 Summary Score in Patients With Hematologic Malignancies. Value in health : the journal of the International Society for Pharmacoeconomics and Outcomes Research. 2019;22(11):1303-10.

37. Giesinger JM, Kieffer JM, Fayers PM, Groenvold M, Petersen MA, Scott NW, et al. Replication and validation of higher order models demonstrated that a summary score for the EORTC QLQ-C30 is robust. J Clin Epidemiol. 2016;69:79-88.

38. Dimopoulos MA, Goldschmidt H, Niesvizky R, Joshua D, Chng WJ, Oriol A, et al. Carfilzomib or bortezomib in relapsed or refractory multiple myeloma (ENDEAVOR): an interim overall survival analysis of an open-label, randomised, phase 3 trial. The Lancet Oncology. 2017;18(10):1327-37.

39. Dimopoulos MA, Moreau P, Palumbo A, Joshua D, Pour L, Hajek R, et al. Carfilzomib and dexamethasone versus bortezomib and dexamethasone for patients with relapsed or refractory multiple myeloma (ENDEAVOR): a randomised, phase 3, open-label, multicentre study. The Lancet Oncology. 2016;17(1):27-38.

40. Ludwig H, Moreau P, Dimopoulos MA, Mateos MV, Kaiser M, Hajek R, et al. Health-related quality of life in the ENDEAVOR study: carfilzomib-dexamethasone vs bortezomib-dexamethasone in relapsed/refractory multiple myeloma. Blood cancer journal. 2019;9(3):23.

41. Stewart AK, Dimopoulos MA, Masszi T, Spicka I, Oriol A, Hajek R, et al. Health-Related Quality of Life Results From the Open-Label, Randomized, Phase III ASPIRE Trial Evaluating Carfilzomib, Lenalidomide, and Dexamethasone Versus Lenalidomide and Dexamethasone in Patients With Relapsed Multiple Myeloma. Journal of clinical oncology. 2016.

42. Stewart AK, Rajkumar SV, Dimopoulos MA, Masszi T, Spicka I, Oriol A, et al. Carfilzomib, lenalidomide, and dexamethasone for relapsed multiple myeloma. The New England journal of medicine. 2015;372(2):142-52.

43. Jakubowiak AJ, Dytfeld D, Griffith KA, Lebovic D, Vesole DH, Jagannath S, et al. A phase 1/2 study of carfilzomib in combination with lenalidomide and low-dose dexamethasone as a frontline treatment for multiple myeloma. Blood. 2012;120(9):1801-9.

44. Mushtaq A, Kapoor V, Latif A, Iftikhar A, Zahid U, McBride A, et al. Efficacy and toxicity profile of carfilzomib based regimens for treatment of multiple myeloma: A systematic review. Critical reviews in oncology/hematology. 2018;125:1-11.

45. King T, Faiman B. Steroid-Associated Side Effects: A Symptom Management Update on Multiple Myeloma Treatment. Clinical journal of oncology nursing. 2017;21(2):240-9.

46. Fayers P, Aaronson, N. K., Bjordal, K., Groenvold, M., Curran, D., & Bottomley, A. . The EORTC QLQ-C30 Scoring Manual (3rd Edition). Brussels: European Organisation for Research and Treatment of Cancer. 2001.

47. Administration and Scoring Guidelines [www.facit.org](file:///\\rsyd.net\Users\mtking\Library\Mobile%20Documents\com~apple~CloudDocs\Documents\Mad\Collaborations\Current\Danish_CARFI%20trial\CARFI%20QOL%20paper%202023\04.CARFI%20HRQL%20manuscript%20-%20Journal%20of%20Patient-Reported%20Outcomes%20-%20JPRO-D-23-00191\www.facit.org): Functional Assessment of Chronic Illness Therapy; [FACT/GOG-Ntx Scoring Guidelines (Version 4)

48. Osoba D, Bezjak A, Brundage M, Zee B, Tu D, Pater J, et al. Analysis and interpretation of health-related quality-of-life data from clinical trials: basic approach of The National Cancer Institute of Canada Clinical Trials Group. Eur J Cancer. 2005;41(2):280-7.

49. Ringash J, O'Sullivan B, Bezjak A, Redelmeier DA. Interpreting clinically significant changes in patient-reported outcomes. Cancer. 2007;110(1):196-202.

50. Cocks K, King MT, Velikova G, Martyn St-James M, Fayers PM, Brown JM. Evidence-based guidelines for determination of sample size and interpretation of the European Organisation for the Research and Treatment of Cancer Quality of Life Questionnaire Core 30. J Clin Oncol. 2011;29(1):89-96.

51. Cocks K, King MT, Velikova G, de Castro G, Jr., Martyn St-James M, Fayers PM, et al. Evidence-based guidelines for interpreting change scores for the European Organisation for the Research and Treatment of Cancer Quality of Life Questionnaire Core 30. Eur J Cancer. 2012;48(11):1713-21.

52. Sully K, Trigg A, Bonner N, Moreno-Koehler A, Trennery C, Shah N, et al. Estimation of minimally important differences and responder definitions for EORTC QLQ-MY20 scores in multiple myeloma patients. Eur J Haematol. 2019;103(5):500-9.

53. Yost KJ, Cella D, Chawla A, Holmgren E, Eton DT, Ayanian JZ, et al. Minimally important differences were estimated for the Functional Assessment of Cancer Therapy-Colorectal (FACT-C) instrument using a combination of distribution- and anchor-based approaches. J Clin Epidemiol. 2005;58(12):1241-51.

54. Calhoun EA, Welshman EE, Chang CH, Lurain JR, Fishman DA, Hunt TL, et al. Psychometric evaluation of the Functional Assessment of Cancer Therapy/Gynecologic Oncology Group-Neurotoxicity (Fact/GOG-Ntx) questionnaire for patients receiving systemic chemotherapy. Int J Gynecol Cancer. 2003;13(6):741-8.

55. Kemmler G, Zabernigg A, Gattringer K, Rumpold G, Giesinger J, Sperner-Unterweger B, et al. A new approach to combining clinical relevance and statistical significance for evaluation of quality of life changes in the individual patient. J Clin Epidemiol. 2010;63(2):171-9.

56. Johnsen AT, Tholstrup D, Petersen MA, Pedersen L, Groenvold M. Health related quality of life in a nationally representative sample of haematological patients. European journal of haematology. 2009;83(2):139-48.

57. King MT, Viney R, Simon Pickard A, Rowen D, Aaronson NK, Brazier JE, et al. Australian Utility Weights for the EORTC QLU-C10D, a Multi-Attribute Utility Instrument Derived from the Cancer-Specific Quality of Life Questionnaire, EORTC QLQ-C30. Pharmacoeconomics. 2018;36(2):225-38.

58. Kemmler G, Gamper E, Nerich V, Norman R, Viney R, Holzner B, et al. German value sets for the EORTC QLU-C10D, a cancer-specific utility instrument based on the EORTC QLQ-C30. Qual Life Res. 2019;28(12):3197-211.

59. King MT, Costa DS, Aaronson NK, Brazier JE, Cella DF, Fayers PM, et al. QLU-C10D: a health state classification system for a multi-attribute utility measure based on the EORTC QLQ-C30. Qual Life Res. 2016;25(3):625-36.

60. Calvert M, Kyte D, Mercieca-Bebber R, Slade A, Chan AW, King MT, et al. Guidelines for Inclusion of Patient-Reported Outcomes in Clinical Trial Protocols: The SPIRIT-PRO Extension. The Journal of the American Medical Association. 2018;319(5):483-94.

61. Kvam AK, Wisloff F, Fayers PM. Minimal important differences and response shift in health-related quality of life; a longitudinal study in patients with multiple myeloma. Health and quality of life outcomes. 2010;8:79-7525-8-79.
