# Supplementary material for: Health-related quality of life and quality-adjusted progression free survival for carfilzomib and dexamethasone maintenance following salvage autologous stem-cell transplantation in patients with multiple myeloma: a randomized phase 2 trial by the Nordic Myeloma Study Group
Source: J Patient Rep Outcomes. 2024 Feb 5;8:15. doi: 10.1186/s41687-024-00691-2 (PMC10844184; doi:10.1186/s41687-024-00691-2)
Supplement: Supplementary file 3 — Supplementary Material 3 [file 41687_2024_691_MOESM3_ESM.docx]

**Supplementary appendix**

**Health-related quality of life and quality-adjusted progression-free-survival for carfilzomib and dexamethasone maintenance following salvage autologous stem-cell transplantation:** A randomized phase 2 trial by the Nordic Myeloma Study Group

# **Table S1. ~~Questionnaire~~ Patient-reported outcomes completion rate.**

~~Questionnaire~~ Patient-reported outcomes (PRO) completion rates were assessed at each PRO assessment time point as the proportion of patients on protocol with completed questionnaires (enough completed item to calculate the ~~domain of~~ EORTC QLQ-C30 Summary score ~~physical functioning~~) of the number of patients expected to complete (on protocol).

|  | **Kd maintenance (n=82)** | | **Observation**  **(n=86)** | | **Total**  **(n=168)** | |
| --- | --- | --- | --- | --- | --- | --- |
|  | **Number of forms** | | **Number of forms** | | **Number of forms** | |
| **Month** | Expected | Completed | Expected | Completed | Expected | Completed |
| 0 | 80 | 76 | 86 | 76 | 166 | 152 |
| 2 | 75 | 69 | 84 | 74 | 159 | 143 |
| 4 | 73 | 68 | 82 | 77 | 155 | 145 |
| 6 | 72 | 71 | 77 | 71 | 149 | 142 |
| 8 | 66 | 62 | 69 | 64 | 135 | 126 |
| 10 | 62 | 60 | 61 | 58 | 123 | 118 |
| 12 | 55 | 54 | 51 | 48 | 106 | 102 |
| 14 | 51 | 47 | 44 | 35 | 95 | 82 |
| 16 | 42 | 41 | 35 | 31 | 77 | 72 |
| 18 | 33 | 33 | 30 | 25 | 63 | 58 |
| 20 | 27 | 27 | 25 | 21 | 52 | 48 |
| 22 | 22 | 22 | 18 | 16 | 40 | 38 |
| **Total** | **658** | **630** | **662** | **596** | **1320** | **1226** |
| **Kd,** carfilzomib-dexamethasone; Expected=on protocol at each specific assessment time point | | | | | | |

**Randomized (p=168)**

**Allocated to Kd maintenance (p=82)**

**Allocated to observation (p=86)**

Drop out (p=2)

- Withdrawn consent (p=2)

**On protocol (p=80)**

**Completed questionnaires (q= 76)**

**On protocol (p=86)**

**Completed questionnaires (q=76)**

**0 months**

Drop out (p=5)

- Withdrawn consent (p=2)

- Progressive disease (p=1)

- Neutropenia (p=1)

- Doctors decision (p=1)

Drop out (p=2)

- Withdrawn consent (p=1)

- Progressive disease (p=1)

**On protocol (p=75)**

**Completed questionnaires (q= 69)**

**On protocol (p=84)**

**Completed questionnaires (q=74)**

Drop out (p=2)

- Progressive disease (p=2)

**2 months**

Drop out (p=1)

- Withdrawn consent (p=1)

Drop out (p=2)

- Progressive disease (p=2)

**On protocol (p=82)**

**Completed questionnaires (q= 77)**

**On protocol (p=73)**

**Completed questionnaires (q=68)**

**4 months**

Drop out (p=5)

- Progressive disease (p=5)

**On protocol (p=77)**

**Completed questionnaires (q=71)**

**On protocol (p=72)**

**Completed questionnaires (q= 71)**

**6 months**

Drop out (p=8)

- Progressive disease (p=7)

- Termination of study (p=1)

Drop out (p=6)

- Progressive disease (p=6)

**8 months**

**On protocol (p=69)**

**Completed questionnaires (q= 64)**

**On protocol (p=66)**

**Completed questionnaires (q= 62)**

**Continued at the next page**

Drop out (p=4)

- Withdrawn consent (p=1)

- Progressive disease (p=1)

- Termination of study (p=2)

Drop out (p=8)

- Progressive disease (p=4)

- Termination of study (p=4)

**On protocol (p=62)**

**Completed questionnaires (q=60)**

**On protocol (p=61)**

**Completed questionnaires (q=58)**

**10 months**

Drop out (p=10)

- Progressive disease (p=4)

- Termination of study (p=6)

Drop out (p=7)

- Death (p=1)

- Termination of study (p=6)

-

..

**12 months**

**On protocol (p=51)**

**Completed questionnaires (q=48)**

**On protocol (p=55)**

**Completed questionnaires (q=54)**

Drop out (p=7)

- Progressive disease (p=4)

- Termination of study (p=3)

Drop out (p=4)

- Progressive disease (p=2)

- Termination of study (p=2)

**On protocol (p=51)**

**Completed questionnaires (q=47)**

**14 months**

**On protocol (p=44)**

**Completed questionnaires (q=35)**

Drop out (p=9)

- Progressive disease (p=4)

- Termination of study (p=4)

- Another cancer (p=1)

Drop out (p=9)

- Progressive disease (p=6)

- Termination of study (p=3)

**On protocol (p=35)**

**Completed questionnaires (q=31)**

**16 months**

**On protocol (p=42)**

**Completed questionnaires (q=41)**

Drop out (p=5)

- Progressive disease (p=5)

Drop out (p=9)

- Progressive disease (p=4)

- Termination of study (p=5)

..

**On protocol (p=33)**

**Completed questionnaires (q=33)**

**On protocol (p=30)**

**Completed questionnaires (q=25)**

**18 months**

**Continued at the next page**

Drop out (p=6)

- Withdrawn consent (P=1)

- Progressive disease (p=2)

- Termination of study (p=3)

-

..

Drop out (p=5)

- Progressive disease (p=3)

- Termination of study (p=2)

**On protocol (p=27)**

**Completed questionnaires (q=27)**

**On protocol (p=25)**

**Completed questionnaires (q=21)**

**20 months**

Drop out (p=5)

- Progressive disease (p=3)

- Termination of study (p=2)

Drop out (p=7)

- Progressive disease (p=6)

- Termination of study (p=1)

**On protocol (p=18)**

**Completed questionnaires (q=16)**

**On protocol (p=22)**

**Completed questionnaires (q=22)**

**22 months**

The last follow-up time point for analysis, interpretation and presentation of result was defined as the time point *before* ~~the time point where~~ the number of patients on protocol in one of the groups became ~~is~~ less than 15.

# **Figure S1. CONSORT flow diagram until last follow-up time point**

**Table S2. EORTC QLQ-C30 Summary score.** Within group change from randomization and between groups differences of EORTC QLQ-C30 Summary score. Statistically significant estimates (p<0.05 for months 2-8 and p<0.01 for 10 months and onwards) that also reached the minimal important difference of 10 for within group changes or between group differences are marked in **bold**.

| **QLQ-C30-sum score** | **Kd maintenance group** | | | **Observation group** | | | **Between groups** | |
| --- | --- | --- | --- | --- | --- | --- | --- | --- |
|  | **Mean score per visit** | **Mean score change (95%CI)** | **p-value** | **Mean score per visit** | **Mean score change (95%CI)** | **p-value** | **Mean score difference (95%CI)** | **p-value** |
| **Month** |  |  |  |  |  |  |  |  |
| **0** | 79.28 | - | - | 78.05 | - | - | - | - |
| **2** | 82.69 | 3.86  (1.82; 5.89) | <0.001 | 82.93 | 4.79  (2.34; 7.23) | <0.001 | -0.93  (-4.08; 2.22) | 0.562 |
| **4** | 82.26 | 2.63  (0.40; 4.87) | 0.021 | 84.42 | 6.13  (3.57; 8.69) | <0.001 | -3.50  (-6.90; -0.09) | 0.044 |
| **6** | 82.79 | 2.77  (-0.24; 5.77) | 0.071 | 83.68 | 5.04  (2.24: 7.85) | <0.001 | -2.28  (-6.40; 1.85) | 0.279 |
| **8** | 82.43 | 1.24  (-2.06; 4.54) | 0.461 | 83.75 | 5.86  (3.17; 8.56) | <0.001 | -4.62  (-8.86: -0.39) | 0.032 |
| **10** | 84.91 | 3.14  (0.70; 5.57) | 0.012 | 84.01 | 5.37  (1.98; 8.76) | 0.002 | -2.23  (-6.34; 1.87) | 0.286 |
| **12** | 86.43 | 4.27  (1.66; 6.88) | 0.001 | 83.51 | 5.19  (2.49; 7.90) | <0.001 | -0.92  (-4.67; 2.82) | 0.628 |
| **14** | 84.62 | 2.63  (0.12; 5.14) | 0.040 | 82.80 | 6.31  (3.72; 8.89) | <0.001 | -3.67  (-7.26; -0.09) | 0.045 |
| **16** | 85.71 | 3.80  (1.33; 6.27) | 0.003 | 84.40 | 7.32  (4.67; 9.98) | <0.001 | -3.52  (-7.12; 0.08) | 0.055 |
| **18** | 85.11 | 2.77  (-0.27; 5.80) | 0.074 | 83.08 | 5.66  (0.41; 10.91) | 0.035 | -2.89  (-8.96; 3.18) | 0.350 |
| **20** | 85.63 | 3.61  (0.86; 6.36) | 0.010 | 81.79 | 5.16  (1.58; 8.74) | 0.005 | -1.55  (-6.04; 2.94) | 0.498 |
| **22** | 85.84 | 3.32  (-0.06; 6.70) | 0.054 | 83.30 | 5.90  (2.56; 9.23) | 0.001 | -2.58  (-7.26; 2.11) | 0.282 |

Kd; Carfilzomib-dexamethsone, CI; Confidence interval

**Table S3. Physical functioning.** Within group change from randomization and between groups differences of EORTC QLQ-C30 physical functioning. Statistically significant estimates (p<0.01) that also reached the minimal important difference for within group changes or between group differences of 2 points are marked in **bold**.

| **Physical functioning** | **Kd maintenance group** | | | **Observation group** | | | **Between groups** | |
| --- | --- | --- | --- | --- | --- | --- | --- | --- |
|  | **Mean score per visit** | **Mean score change (95%CI)** | **p-value** | **Mean score per visit** | **Mean score change (95%CI)** | **p-value** | **Mean score difference (95%CI)** | **p-value** |
| **Month** |  |  |  |  |  |  |  |  |
| **0** | 77.41 |  | - | 75.90 | - | - | - | - |
| **2** | 83.43 | **5.79**  **(2.93; 8.65)** | **<0.001** | 79.53 | **3.98**  **(1.03; 6.93)** | **0.008** | 1.81  (-2.37; 5.99) | 0.396 |
| **4** | 82.08 | 3.65  (0.29; 7.00) | 0.033 | 80.26 | **4.98**  **(1.51; 8.46)** | **0.005** | -1.34  (-6.22; 3.55) | 0.592 |
| **6** | 82.82 | **4.46**  **(1.22; 7.70)** | **0.007** | 79.12 | **3.81**  **(-0.06; 7.68)** | **0.054** | 0.65  (-4.45; 5.75) | 0.803 |
| **8** | 80.72 | 1.13  (-3.43; 5.69) | 0.628 | 80.91 | **6.47**  **(3.05; 9.89)** | **<0.001** | -5.34  (-10.94; 0.26) | 0.062 |
| **10** | 84.31 | 3.86  (-0.09; 7.81) | 0.056 | 81.03 | **5.85**  **(2.17; 9.53)** | **0.002** | -1.99  (-7.31; 3.32) | 0.462 |
| **12** | 85.68 | 4.52  (0.33; 8.70) | 0.034 | 79.55 | **5.73**  **(1.81; 9.66)** | **0.004** | -1.22  (-6.89; 4.46) | 0.674 |
| **14** | 85.83 | **5.38**  **(1.70; 9.07)** | **0.004** | 79.68 | **7.28**  **(2.55; 12.01)** | **0.003** | -1.90  (-7.85; 4.05) | 0.532 |
| **16** | 86.34 | **5.38**  **(2.20; 8.56)** | **0.001** | 76.56 | 4.76  (0.41; 9.11) | 0.032 | 0.62  (-4.75; 5.98) | 0.821 |
| **18** | 86.26 | 4.69  (0.80; 8.58) | 0.018 | 77.33 | 5.49  (-1.55; 12.53) | 0.126 | -0.80  (-8.78; 7.18) | 0.844 |
| **20** | 85.93 | **5.20**  **(1.30; 9.09)** | **0.009** | 74.29 | 2.83  (-2.41; 8.06) | 0.290 | 2.37  (-4.02; 8.76) | 0.468 |
| **22** | 86.59 | **6.65**  **(2.91; 10.40)** | **<0.001** | 75.42 | 3.44  (-3.00; 9.88) | 0.295 | 3.22  (-4.19; 10.63) | 0.395 |

Kd; Carfilzomib-dexamethsone, CI; Confidence interval

**Table S4. Role functioning.** Within group change from randomization and between groups differences of EORTC QLQ-C30 role functioning. Statistically significant estimates (p<0.01) that also reached the minimal important difference for within group changes or between group differences of 6 points are marked in **bold**.

| **Role functioning** | **Kd maintenance group** | | | **Observation group** | | | **Between groups** | |
| --- | --- | --- | --- | --- | --- | --- | --- | --- |
|  | **Mean score per visit** | **Mean score change (95%CI)** | **p-value** | **Mean score per visit** | **Mean score change (95%CI)** | **p-value** | **Mean score difference (95%CI)** | **p-value** |
| **Month** |  |  |  |  |  |  |  |  |
| **0** | 65.60 | - | - | 63.03 | - | - | - | - |
| **2** | 74.29 | **9.67**  **(3.07; 16.27)** | **0.004** | 72.29 | **9.09**  **(2.78; 15.40)** | **0.005** | 0.58  (-8.48; 9.65) | 0.900 |
| **4** | 75.60 | **9.23**  **(3.81; 14.65)** | **0.001** | 77.71 | **14.02**  **(7.89; 20.16)** | **<0.001** | -4.79  (-12.91; 3.33) | 0.247 |
| **6** | 74.18 | 7.82  (0.72; 14.93) | 0.031 | 75.00 | **11.54**  **(4.53; 18.54)** | **0.001** | -3.71  (-13.76; 6.33) | 0.469 |
| **8** | 75.64 | 7.74  (0.23; 15.25) | 0.043 | 74.48 | **12.18**  **(5.39; 18.96)** | **<0.001** | -4.44  (-14.52; 5.65) | 0.389 |
| **10** | 76.94 | **8.00**  **(2.44; 13.57)** | **0.005** | 77.30 | **13.37**  **(5.21; 21.52)** | **0.001** | -5.36  (-15.15; 4.42) | 0.283 |
| **12** | 79.63 | **10.66**  **(2.90; 18.42)** | **0.007** | 75.69 | **12.26**  **(5.29; 19.23)** | **0.001** | -1.60  (-12.13; 8.93) | 0.766 |
| **14** | 75.69 | 6.97  (0.11; 13.84) | 0.046 | 70.83 | **10.60**  **(2.98; 18.22)** | **0.006** | -3.62  (-13.90; 6.65) | 0.489 |
| **16** | 83.33 | **12.99**  **(6.28; 19.69)** | **<0.001** | 76.34 | **16.36**  **(8.40; 24.32)** | **<0.001** | -3.38  (-13.82; 7.07) | 0.526 |
| **18** | 79.29 | 6.93  (0.47; 13.38) | 0.036 | 73.33 | 11.23  (0.16; 22.30) | 0.047 | -4.31  (-17.48; 8.87) | 0.522 |
| **20** | 81.48 | **10.40**  **(2.70; 18.09)** | **0.008** | 70.63 | 10.62  (0.56; 20.67) | 0.039 | -0.22  (-12.84; 12.40) | 0.973 |
| **22** | 83.33 | 10.12  (0.60; 19.64) | 0.037 | 70.83 | 9.18  (-0.11; 18.46) | 0.053 | 0.94  (-12.39; 14.28) | 0.890 |

Kd; Carfilzomib-dexamethsone, CI; Confidence interval

**Table S5. Social functioning.** Within group change and between groups differences of EORTC QLQ-C30 social functioning. Statistically significant estimates (p<0.01) that also reached the minimal important difference for within group changes or between group differences of 3 points are marked in **bold**.

| **Social functioning** | **Kd maintenance group** | | | **Observation group** | | | **Between groups** | |
| --- | --- | --- | --- | --- | --- | --- | --- | --- |
|  | **Mean score per visit** | **Mean score change (95%CI)** | **p-value** | **Mean score per visit** | **Mean score change (95%CI)** | **p-value** | **Mean score difference (95%CI)** | **p-value** |
| **Month** |  |  |  |  |  |  |  |  |
| **0** | 74.15 |  | - | 73.59 | - | - | - | - |
| **2** | 81.67 | **6.82**  **(2.20; 11.44)** | **0.004** | 82.25 | **8.25**  **(3.58; 12.93)** | **0.001** | -1.43  (-7.95; 5.08) | 0.666 |
| **4** | 80.92 | 5.31  (0.58; 10.04) | 0.028 | 83.33 | **8.90**  **(4.22; 13.58)** | **<0.001** | -3.59  (-10.32; 3.13) | 0.295 |
| **6** | 83.10 | **7.40**  **(1.87; 12.94)** | **0.009** | 82.41 | **7.77**  **(3.37; 12.17)** | **0.001** | -0.37  (-7.35; 6.61) | 0.917 |
| **8** | 81.25 | 3.81  (-1.67; 9.28) | 0.173 | 83.07 | **9.34**  **(5.07; 13.62)** | **<0.001** | -5.54  (-12.41; 1.34) | 0.114 |
| **10** | 83.33 | 5.62  (-0.11; 11.34) | 0.054 | 80.46 | 5.12  (-1.29; 11.53) | 0.118 | 0.50  (-8.09; 9.08) | 0.910 |
| **12** | 85.80 | **7.87**  **(2.43; 13.31)** | **0.005** | 80.56 | 5.52  (0.67; 10.36) | 0.026 | 2.35  (-4.86; 9.56) | 0.522 |
| **14** | 82.99 | 5.17  (0.20; 10.14) | 0.042 | 78.10 | 6.50  (1.11; 11.90) | 0.018 | -1.34  (-8.56; 5.89) | 0.717 |
| **16** | 81.30 | 2.95  (-2.45; 8.36) | 0.285 | 82.26 | **9.14**  **(3.09; 15.19)** | **0.003** | -6.19  (-14.28; 1.90) | 0.134 |
| **18** | 82.32 | 2.78  (-2.73; 8.29) | 0.322 | 80.00 | 6.31  (-0.82; 13.45) | 0.083 | -3.53  (-12.46; 5.40) | 0.438 |
| **20** | 84.57 | 4.87  (-1.39; 11.12) | 0.128 | 74.60 | 3.42  (-3.12; 9.96) | 0.306 | 1.45  (-7.67; 10.57) | 0.755 |
| **22** | 88.64 | **6.86**  **(1.90; 11.82)** | **0.007** | 80.21 | 7.33  (-0.74; 15.40) | 0.075 | -0.47  (-9.89: 8.95) | 0.922 |

Kd; Carfilzomib-dexamethsone, CI; Confidence interval

**Table S6. Appetite loss.** Within group change from randomization and between groups differences of EORTC QLQ-C30 appetite loss. Statistically significant estimates (p<0.01) that also reached the minimal important difference for within group changes or between group differences of 7 points are marked in **bold**.

| **Appetite loss** | **Kd maintenance group** | | | **Observation group** | | | **Between groups** | |
| --- | --- | --- | --- | --- | --- | --- | --- | --- |
|  | **Mean score per visit** | **Mean score change (95%CI)** | **p-value** | **Mean score per visit** | **Mean score change (95%CI)** | **p-value** | **Mean score difference (95%CI)** | **p-value** |
| **Month** |  |  |  |  |  |  |  |  |
| **0** | 12.99 |  | - | 16.24 | - | - | - | - |
| **2** | 7.62 | -6.60  (-11.56; -1.63) | 0.009 | 7.79 | **-8.46**  **(-13.72; -3.20)** | **0.002** | 1.86  (-5.22; 8.94) | 0.607 |
| **4** | 6.28 | **-7.07**  **(-12.18; -1.97)** | **0.007** | 8.66 | **-8.24**  **(-14.49; -1.98)** | **0.010** | 1.16  (-6.65; 8.98) | 0.771 |
| **6** | 10.80 | -2.06  (-9.23; 5.12) | 0.574 | 6.02 | **-10.80**  **(-16.50; -5.09)** | **<0.001** | 8.74  (-0.26; 17.74) | 0.057 |
| **8** | 10.94 | -1.11  (-8.21; 5.99) | 0.758 | 4.17 | **-12.87**  **(-19.21; -6.52)** | **<0.001** | 11.75  (2.47; 21.04) | 0.013 |
| **10** | 4.44 | -6.81  (-12.53; -1.09) | 0.020 | 3.45 | **-12.48**  **(-19.05; -5.92)** | **<0.001** | 5.67  (-2.81; 14.16) | 0.190 |
| **12** | 2.47 | **-8.58**  **(-13.62; -3.54)** | **0.001** | 2.78 | **-13.22**  **(-18.63; -7.80)** | **<0.001** | 4.64  (-2.53; 11.81) | 0.205 |
| **14** | 4.86 | **-6.27**  **(-12.27; -0.26)** | **0.041** | 2.78 | **-13.82**  **(-21.15; -6.49)** | **<0.001** | 7.55  (-1.46; 16.57) | 0.100 |
| **16** | 4.07 | **-7.18**  **(-12.24; -2.12)** | **0.005** | 1.08 | **-15.17**  **(-21.93; -8.42)** | **<0.001** | 8.00  (-0.22; 16.21) | 0.056 |
| **18** | 8.08 | -2.46  (-9.47; 4.56) | 0.492 | 5.33 | -10.96  (-20.18; -1.75) | 0.020 | 8.51  (-3.25; 20.26) | 0.156 |
| **20** | 3.70 | -6.79  (-12.50; -1.09) | 0.020 | 1.59 | **-15.10**  **(-21.98; -8.23)** | **<0.001** | 8.31  (-0.38; 17.01) | 0.061 |
| **22** | 4.55 | -6.26  (-12.80; 0.29) | 0.061 | 2.80 | **-15.30**  **(-23.01; -7.59)** | **<0.001** | 9.05  (-0.91; 19.01) | 0.075 |

Kd; Carfilzomib-dexamethsone, CI; Confidence interval

**Table S7. Fatigue.** Within group change from randomization and between groups differences of EORTC QLQ-C30 fatigue. Statistically significant estimates (p<0.01) that also reached the minimal important difference for within group changes or between group differences of 4 points are marked in **bold**.

| **Fatigue** | **Kd maintenance group** | | | **Observation group** | | | **Between groups** | |
| --- | --- | --- | --- | --- | --- | --- | --- | --- |
|  | **Mean score per visit** | **Mean score change (95%CI)** | **p-value** | **Mean score per visit** | **Mean score change (95%CI)** | **p-value** | **Mean score difference (95%CI)** | **p-value** |
| **Month** |  |  |  |  |  |  |  |  |
| **0** | 35.61 |  | - | 35.61 | - | - | - | - |
| **2** | 31.80 | -3.90  (-8.14; 0.33) | 0.071 | 28.13 | **-7.27**  **(-11.67; -2.87)** | **0.001** | 3.37  (-2.69; 9.42) | 0.276 |
| **4** | 29.15 | -5.97  (-10.81; -1.14) | 0.015 | 25.25 | **-9.87**  **(-14.56; -5.19)** | **<0.001** | 3.90  (-2.85; 10.65) | 0.258 |
| **6** | 30.67 | -3.16  (-8.81; 2.49) | 0.273 | 27.31 | **-7.04**  **(-11.82; -2.25)** | **0.004** | 3.87  (-3.47; 11.22) | 0.301 |
| **8** | 31.45 | -2.53  (-8.30; 3.24) | 0.390 | 26.56 | **-9.09**  **(-13.61; -4.57)** | **<0.001** | 6.56  (-0.72; 13.84) | 0.078 |
| **10** | 27.41 | -4.76  (-9.84; 0.33) | 0.067 | 27.97 | -7.09  (-13.38; -0.80) | 0.027 | 2.33  (-5.75; 10.41) | 0.571 |
| **12** | 24.49 | -6.28  (-11.85; -0.71) | 0.027 | 26.27 | **-8.66**  **(-13.30; -4.01)** | **<0.001** | 2.38  (-4.82; 9.58) | 0.517 |
| **14** | 27.55 | -4.50  (-9.30; 0.31) | 0.067 | 25.00 | **-11.27**  **(-16.22; -6.33)** | **<0.001** | 6.78  (-0.10; 13.66) | 0.053 |
| **16** | 24.93 | **-6.93**  **(-11.38; -2.48)** | **0.002** | 29.03 | **-7.44**  **(-12.73; -2.15)** | **0.006** | 0.51  (-6.45; 7.47) | 0.886 |
| **18** | 28.28 | -3.92  (-9.17; 1.34) | 0.144 | 26.67 | **-9.89**  **(-16.54; -3.24)** | **0.004** | 5.97  (-2.67; 14.61) | 0.176 |
| **20** | 25.51 | -7.04  (-13.02; -1.06) | 0.021 | 29.63 | -6.69  (-13.82; 0.45) | 0.066 | -0.35  (-9.74; 9.03) | 0.941 |
| **22** | 26.77 | -7.17  (-15.15; 0.81) | 0.078 | 28.47 | -6.64  (-12.73; -0.55) | 0.033 | -0.53  (-10.59; 9.52) | 0.917 |

Kd; Carfilzomib-dexamethsone, CI; Confidence interval

**Table S8. Body image.** Within group change from randomization and between groups differences of EORTC QLQ-MY20 body image. Statistically significant estimates (p<0.01) that also reached the minimal important difference for within group changes or between group differences of 13 points are marked in **bold**.

| **Body image** | **Kd maintenance group** | | | **Observation group** | | | **Between groups** | |
| --- | --- | --- | --- | --- | --- | --- | --- | --- |
|  | **Mean score per visit** | **Mean score change (95%CI)** | **p-value** | **Mean score per visit** | **Mean score change (95%CI)** | **p-value** | **Mean score difference (95%CI)** | **p-value** |
| **Month** |  |  |  |  |  |  |  |  |
| **0** | 68.42 | - | - | 70.22 | - | - | - | - |
| **2** | 75.36 | 8.87  (1.62; 16.11) | 0.016 | 80.95 | 11.02  (5.56; 16.47) | <0.001 | -2.15  (-11.34; 7.04) | 0.647 |
| **4** | 78.10 | 10.01  (4.63; 15.40) | <0.001 | 79.39 | 8.97  (3.64; 14.29) | 0.001 | 1.05  (-6.62; 8.71) | 0.789 |
| **6** | 80.88 | 10.65  (4.88; 16.42) | <0.001 | 81.48 | 12.07  (6.08; 18.05) | <0.001 | -1.42  (-9.79; 6.96) | 0.740 |
| **8** | 78.13 | 8.24  (0.90; 15.58) | 0.028 | 82.29 | **13.60**  **(7.05; 20.14)** | **<0.001** | -5.36  (-15.30; 4.59) | 0.291 |
| **10** | 79.44 | 8.52  (2.37; 14.67) | 0.007 | 81.03 | 11.00  (5.39; 16.62) | <0.001 | -2.48  (-10.77; 5.80) | 0.557 |
| **12** | 82.39 | 11.00  (4.54; 17.46) | 0.001 | 83.33 | **13.92**  **(8.03; 19.80)** | **<0.001** | -2.92  (-11.55; 5.71) | 0.507 |
| **14** | 79.43 | 9.88  (2.25; 17.51) | 0.011 | 79.63 | **14.21**  **(7.38; 21.05)** | **<0.001** | -4.33  (-14.68; 6.02) | 0.412 |
| **16** | 80.83 | 12.17  (4.69; 19.66) | 0.001 | 79.57 | 12.55  (5.72; 19.37) | <0.001 | -0.38  (-10.51; 9.76) | 0.942 |
| **18** | 82.29 | **14.42**  **(7.00; 21.83)** | **<0.001** | 74.67 | 8.25  (-3.28; 19.78) | 0.161 | 6.17  (-7.54; 19.87) | 0.378 |
| **20** | 86.42 | **17.45**  **(10.29; 24.61)** | **<0.001** | 69.84 | 7.33  (-4.58; 19.25) | 0.228 | 10.12  (-3.56; 23.79) | 0.147 |
| **22** | 83.33 | **13.72**  **(5.66; 21.78)** | **0.001** | 64.58 | 2.12  (-10.24; 14.49) | 0.737 | 11.59  (-3.13; 26.32) | 0.123 |

Kd; Carfilzomib-dexamethsone, CI; Confidence interval

**Table S9. The proportions of patients who improved, remained stable or worsened** in the GHS/QoL and functional domains (except cognitive functioning) and in the QLQ-MY20 Body image domain from randomization to two, four, six and eight months follow-up.

| **Domain** | **Direction** | **Follow-up** | **Kd maintenance** | **Observation** | **Odds ratio (95%CI)** | **P-value** |
| --- | --- | --- | --- | --- | --- | --- |
| GHS/QoL |  | Month | Patients (%) | Patients (%) |  |  |
|  | Improved | 2 | 8 (11.8%) | 7 (9.9%) | 1.22 (0.42-3.57) | 0.717 |
|  | Improved | 4 | 8 (11.9%) | 6 (8.7%) | 1.42 (0.47-4.35) | 0.534 |
|  | Improved | 6 | 11 (15.9%) | 4 (6.2%) | 2.89 (0.87-9.60) | 0.072 |
|  | Improved | 8 | 8 (12.9%) | 8 (13.6%) | 0.94 (0.33-2.70) | 0.915 |
|  | Worsened | 2 | 4 (5.9%) | 5 (7.0%) | 0.82 (0.21-3.21) | 0.781 |
|  | Worsened | 4 | 2 (3.0%) | 6 (8.7%) | 0.32 (0.06-1.66) | 0.157 |
|  | Worsened | 6 | 6 (8.7%) | 6 (9.2%) | 0.94 (0.29-3.07) | 0.914 |
|  | Worsened | 8 | 9 (14.5%) | 7 (11.9%) | 1.26 (0.44-3.64) | 0.667 |
|  | Stable | 2 | 56 (82.4%) | 59 (83.1%) | 0.95 (0.39-2.29) | 0.907 |
|  | Stable | 4 | 57 (85.1%) | 57 (82.6%) | 1.20 (0.48-3.00) | 0.696 |
|  | Stable | 6 | 52 (75.4%) | 55 (84.6%) | 0.56 (0.23-1.33) | 0.182 |
|  | Stable | 8 | 45 (72.6%) | 44 (74.6%) | 0.90 (0.40-2.03) | 0.804 |
| **Domain** | **Direction** | **Follow-up** | **Kd maintenance** | **Observation** | **Odds ratio (95%CI)** | **P-value** |
| Physical functioning |  | Month | Patient (%) | Patients (%) |  |  |
|  | Improved | 2 | 8 (11.8%) | 5 (6.8%) | 1.84 (0.57-5.93) | 0.301 |
|  | Improved | 4 | 4 (6.0%) | 9 (12.5%) | 0.44 (0.13-1.52) | 0.186 |
|  | Improved | 6 | 5 (7.2%) | 8 (11.8%) | 0.59 (0.18-1.89) | 0.367 |
|  | Improved | 8 | 5 (7.9%) | 7 (11.5%) | 0.67 (0.20-2.22) | 0.505 |
|  | Worsened | 2 | 0 (0.0%) | 3 (4.1%) | NA | NA |
|  | Worsened | 4 | 1 (1.5%) | 5 (6.9%) | 0.20 (0.02-1.78) | 0.114 |
|  | Worsened | 6 | 2 (2.9%) | 5 (7.4%) | 0.38 (0.07-2.01) | 0.236 |
|  | Worsened | 8 | 3 (4.8%) | 2 (3.3%) | 1.48 (0.24-9.15) | 0.675 |
|  | Stable | 2 | 60 (88.2%) | 66 (89.2%) | 0.91 (0.32-2.57) | 0.857 |
|  | Stable | 4 | 62 (92.5%) | 58 (80.6%) | 2.99 (1.01-8.83) | **0.040** |
|  | Stable | 6 | 62 (89.9%) | 55 (80.9%) | 2.09 (0.78-5.62) | 0.137 |
|  | Stable | 8 | 55 (87.3%) | 52 (85.2%) | 1.19 (0.43-3.32) | 0.739 |
| **Domain** | **Direction** | **Follow-up** | **Kd maintenance** | **Observation** | **Odds ratio (95%CI)** | **P-value** |
| Role functioning |  | Month | Patient (%) | Patients (%) |  |  |
|  | Improved | 2 | 18 (26.5%) | 26 (35.6%) | 0.65 (0.32-1.34) | 0.242 |
|  | Improved | 4 | 19 (28.4%) | 24 (33.3%) | 0.79 (0.38-1.63) | 0.526 |
|  | Improved | 6 | 17 (24.6%) | 22 (32.4%) | 0.68 (0.32-1.44) | 0.317 |
|  | Improved | 8 | 15 (23.8%) | 19 (31.1%) | 0.69 (0.31-1.53) | 0.360 |
|  | Worsened | 2 | 9 (13.2%) | 8 (11.0%) | 1.24 (0.45-3.42) | 0.678 |
|  | Worsened | 4 | 2 (3.0%) | 4 (5.6%) | 0.52 (0.09-2.95) | 0.456 |
|  | Worsened | 6 | 7 (10.1%) | 7 (10.3%) | 0.98 (0.33-2.97) | 0.977 |
|  | Worsened | 8 | 6 (9.5%) | 4 (6.6%) | 1.50 (0.40-5.60) | 0.544 |
|  | Stable | 2 | 41 (60.3%) | 39 (53.4%) | 1.32 (0.68-2.58) | 0.411 |
|  | Stable | 4 | 46 (68.7%) | 44 (61.1%) | 1.39 (0.69-2.81) | 0.352 |
|  | Stable | 6 | 45 (65.2%) | 39 (57.4%) | 1.39 (0.70-2.78) | 0.345 |
|  | Stable | 8 | 42 (66.7%) | 38 (62.3%) | 1.21 (0.58-2.53) | 0.611 |
| **Domain** | **Direction** | **Follow-up** | **Kd maintenance** | **Observation** | **Odds ratio (95%CI)** | **P-value** |
| Emotional functioning |  | Month | Patient (%) | Patients (%) |  |  |
|  | Improved | 2 | 5 (7.4%) | 7 (9.7%) | 0.74 (0.22-2.44) | 0.617 |
|  | Improved | 4 | 3 (4.5%) | 7 (9.9%) | 0.43 (0.11-1.73) | 0.223 |
|  | Improved | 6 | 6 (8.7%) | 4 (6.0%) | 1.50 (0.40-5.57) | 0.543 |
|  | Improved | 8 | 5 (8.1%) | 3 (5.0%) | 1.67 (0.38-7.30) | 0.494 |
|  | Worsened | 2 | 2 (2.9%) | 2 (2.8%) | 1.06 (0.15-7.75) | 0.954 |
|  | Worsened | 4 | 4 (6.0%) | 2 (2.8%) | 2.19 (0.39-12.37) | 0.364 |
|  | Worsened | 6 | 7 (10.1%) | 2 (3.0%) | 3.67 (0.73-18.35) | 0.093 |
|  | Worsened | 8 | 5 (8.1%) | 5 (8.3%) | 0.96 (0.26-3.52) | 0.957 |
|  | Stable | 2 | 61 (89.7%) | 63 (87.5%) | 1.24 (0.44-3.55) | 0.682 |
|  | Stable | 4 | 60 (89.6%) | 62 (87.3%) | 1.24 (0.44-3.55) | 0.683 |
|  | Stable | 6 | 56 (81.2%) | 61 (91.0%) | 0.42 (0.15-1.19) | 0.096 |
|  | Stable | 8 | 52 (83.9%) | 52 (86.7%) | 0.80 (0.29-2.19) | 0.663 |
| **Domain** | **Direction** | **Follow-up** | **Kd maintenance** | **Observation** | **Odds ratio (95%CI)** | **P-value** |
| Social functioning |  | Month | Patient (%) | Patients (%) |  |  |
|  | Improved | 2 | 15 (22.1%) | 14 (19.4%) | 1.17 (0.52-2.66) | 0.703 |
|  | Improved | 4 | 10 (14.9%) | 15 (21.1%) | 0.65 (0.27-1.58) | 0.344 |
|  | Improved | 6 | 18 (26.1%) | 13 (19.4%) | 1.47 (0.65-3.29) | 0.353 |
|  | Improved | 8 | 12 (19.4%) | 12 (20.0%) | 0.96 (0.39-2.34) | 0.929 |
|  | Worsened | 2 | 2 (2.9%) | 5 (6.9%) | 0.41 (0.08-2.17) | 0.277 |
|  | Worsened | 4 | 6 (9.0%) | 2 (2.8%) | 3.39 (0.66-17.44) | 0.123 |
|  | Worsened | 6 | 4 (5.8%) | 3 (4.5%) | 1.31 (0.28-6.10) | 0.728 |
|  | Worsened | 8 | 8 (12.9%) | 1 (1.7%) | 8.74 (1.06-72.19) | **0.018** |
|  | Stable | 2 | 51 (75.0%) | 53 (73.6%) | 1.08 (0.50-2.30) | 0.851 |
|  | Stable | 4 | 51 (76.1%) | 54 (76.1%) | 1.00 (0.46-2.19) | 0.993 |
|  | Stable | 6 | 47 (68.1%) | 51 (76.1%) | 0.67 (0.31-1.43) | 0.298 |
|  | Stable | 8 | 42 (67.7%) | 47 (78.3%) | 0.58 (0.26-1.31) | 0.188 |
| **Domain** | **Direction** | **Follow-up** | **Kd maintenance** | **Observation** | **Odds ratio (95%CI)** | **P-value** |
| Body image |  | Month | Patient (%) | Patients (%) |  |  |
|  | Improved | 2 | 23 (34.8%) | 21 (30.0%) | 1.25 (0.61-2.56) | 0.546 |
|  | Improved | 4 | 22 (32.8%) | 19 (27.5%) | 1.29 (0.62-2.68) | 0.501 |
|  | Improved | 6 | 19 (29.2%) | 22 (33.8%) | 0.81 (0.38-1.69) | 0.571 |
|  | Improved | 8 | 18 (29.5%) | 22 (37.9%) | 0.68 (0.32-1.47) | 0.331 |
|  | Worsened | 2 | 7 (10.6%) | 3 (4.3%) | 2.65 (0.66-10.71) | 0.158 |
|  | Worsened | 4 | 5 (7.5%) | 5 (7.2%) | 1.03 (0.28-3.74) | 0.961 |
|  | Worsened | 6 | 5 (7.7%) | 4 (6.2%) | 1.27 (0.33-4.96) | 0.730 |
|  | Worsened | 8 | 10 (16.4%) | 6 (10.3%) | 1.70 (0.58-5.02) | 0.334 |
|  | Stable | 2 | 36 (54.5%) | 46 (65.7%) | 0.63 (0.31-1.25) | 0.183 |
|  | Stable | 4 | 40 (59.7%) | 45 (65.2%) | 0.79 (0.39-1.58) | 0.507 |
|  | Stable | 6 | 41 (63.1%) | 39 (60.0%) | 1.14 (0.56-2.31) | 0.718 |
|  | Stable | 8 | 33 (54.1%) | 30 (51.7%) | 1.10 (0.54-2.26) | 0.795 |
| **Domain** | **Direction** | **Follow-up** | **Kd maintenance** | **Observation** | **Odds ratio (95%CI)** | **P-value** |
| Future perspectives |  | Month | Patient (%) | Patients (%) |  |  |
|  | Improved | 2 | 11 (16.4%) | 15 (21.1%) | 0.73 (0.31-1.74) | 0.480 |
|  | Improved | 4 | 12 (17.9%) | 17 (24.3%) | 0.68 (0.30-1.56) | 0.361 |
|  | Improved | 6 | 12 (18.5%) | 19 (28.8%) | 0.56 (0.25-1.27) | 0.164 |
|  | Improved | 8 | 12 (19.7%) | 15 (25.4%) | 0.72 (0.30-1.70) | 0.451 |
|  | Worsened | 2 | 4 (6.0%) | 6 (8.5%) | 0.69 (0.19-2.55) | 0.574 |
|  | Worsened | 4 | 4 (6.0%) | 4 (5.7%) | 1.05 (0.25-4.37) | 0.949 |
|  | Worsened | 6 | 5 (7.7%) | 5 (7.6%) | 1.02 (0.28-3.69) | 0.980 |
|  | Worsened | 8 | 4 (6.6%) | 3 (5.1%) | 1.31 (0.28-6.12) | 0.731 |
|  | Stable | 2 | 52 (77.6%) | 50 (70.4%) | 1.46 (0.68-3.14) | 0.336 |
|  | Stable | 4 | 51 (76.1%) | 49 (70.0%) | 1.37 (0.64-2.92) | 0.420 |
|  | Stable | 6 | 48 (73.8%) | 42 (63.6%) | 1.61 (0.76-3.40) | 0.208 |
|  | Stable | 8 | 45 (73.8%) | 41 (69.5%) | 1.23 (0.56-2.74) | 0.603 |

GHS/QoL; Global Health Scale/Quality of Life, Kd; Carfilzomib maintenance

**Table S10. The average weeks to first recorded improvement**

|  | **Kd maintenance group**  Average weeks to first recorded improvement  (standard deviation) | **Observation group**  Average weeks to first recorded improvement  (standard deviation) |
| --- | --- | --- |
| Global Health scale/QoL | 24.3 (17.9) | 19.3 (11.7) |
| Physical functioning | 24.5 (21.7) | 20.8 (17.6) |
| Role functioning | 15.5 (14.3) | 13.9 (12.1) |
| Emotional functioning | 17.6 (11.8) | 12.8 (10.1) |
| Social functioning | 15.3 (13.6) | 12.2 (6.3) |
| Body image | 16.2 (17.2) | 13.0 (8.6) |
| Future perspectives | 26.7 (21.5) | 18.25 (14.1) |

GHS/QoL; Global Health Scale/Quality of Life, Kd; Carfilzomib maintenance

**Table S11. The proportions of patients with Kd symptoms** defined as fatigue, dyspnea, diarrhea, nausea/vomiting, insomnia, and agitation and restlessness and the items included in the mentioned domains.

| **Domain/**  **item** | **Severity of symptom** | **Kd maintenance**  Proportion of patients with symptoms (%) | **Observation**  Proportion of patients with symptoms (%) | **Odds ratio (95%**  **CI)** | **P-value** |
| --- | --- | --- | --- | --- | --- |
| **Diarrhea** | Mild/moderate/severe | 63% | 62% | 1.01 (0.54; 1.89) | 0.984 |
|  | Moderate/severe | 23% | 24% | 0.88 (0.43; 1.82) | 0.739 |
| **Dyspnoea** | Mild/moderate/severe | 76% | 76% | 0.99 (0.48; 2.03) | 0.973 |
|  | Moderate/severe | 29% | 28% | 1.03 (0.52; 2.02) | 0.942 |
| **Fatigue** | Mild/moderate/severe | 83% | 79% | 1.18 (0.54; 2.58) | 0.681 |
|  | Moderate/severe | 34% | 27% | 1.37 (0.71; 2.67) | 0.350 |
| **Restlessness and agitation** | Mild/moderate/severe | 66% | 51% | 1.90 (1.01; 3.58) | **0.046** |
|  | Moderate/severe | 24% | 16% | 1.61 (0.74; 3.47) | 0.226 |
| **Insomnia** | Mild/moderate/severe | 88% | 72% | 2.60 (1.15; 5.88) | **0.019** |
|  | Moderate/severe | 44% | 38% | 1.23 (0.66; 2.28) | 0.521 |
| **Nausea and vomiting** | Mild/moderate/severe | 34% | 24% | 1.55 (0.79; 3.05) | 0.201 |
|  | Moderate/severe | 6% | 9% | 0.64 (0.20; 2.05) | 0.451 |

GHS/QoL; Global Health Scale/Quality of Life, Kd; Carfilzomib maintenance

# **Table S12. Dose escalation and modification of Kd maintenance therapy**

| **Dose escalation**  Carfilzomib-dexamethsone maintenance therapy consisted of intravenous carfilzomib 27 mg/sqm every second week and oral dexamethasone 20 mg every second week. The maintenance dose of carfilzomib was escalated to 56 mg/sqm after 4 weeks in case of acceptable side effects. |
| --- |
| **Carfilzomib maintenance dose modification**  If a patient experienced ≥Grade 3 neutropenia with fever, Grade 4 neutropenia lasting more than 7 days, platelet count <25 x109/L, or any ≥Grade 3 non-hematologic toxicity considered by the investigator to be related to carfilzomib, then the carfilzomib treatment was suspended. For non-hematologic toxicities, carfilzomib was suspended until the toxicity returns to Grade 2 or better. For hematologic toxicities, carfilzomib was suspended until the subject has an ANC ≥ 0.75 x109/L and a platelet count ≥ 30 x109/L. If, after carfilzomib was suspended and the toxicity did not resolve, as defined above, then study drug had to be discontinued. If the toxicity resolved, as defined above, and carfilzomib was to be restarted, the dose was reduced by one level (going from 56 mg/sqm to 45 mg/sqm, from 45 mg/sqm to 36 mg/sqm or from 36 to 27 mg/sqm. Dose reductions below 27 mg/sqm are not permitted in the maintenance phase. |
| **Dexamethasone dose modification (maintenance phase)**  Dexamethasone dosage is not adjusted due to hematological toxicity.  Steroid induced hyperglycemia was managed by glucose monitoring and insulin administration according to local guidelines. Gastric protection and anti-fungal prophylaxis were administered according to local standards. If the patient experiences side effects grade 3 or 4 considered by the investigator to be related to dexamethasone maintenance treatment, the drug is discontinued for up to four weeks. If the toxicity returns to grade 2 or better, dexamethasone should be restarted at a dose of 10 mg every second week. If the toxicity does not resolve within four weeks or the toxicity returns on the reduced dose of dexamethasone, then this drug must be discontinued permanently. |

.

# **Table S13. Doses of dexamethasone related to maintenance during PRO data collection.**

E.g. at 2 months, the dexamethasone doses administrated at week 2, week 4, week 6 and week 8 are displayed in the table. Therefore the “number of expected dexamethasone doses administrated” is 4-times higher than the number of patients on protocol at each time point.

| **Month, weeks** | **Number of expected dexamethasone doses administered** | **Dexamethasone administered** | | | | | | | |
| --- | --- | --- | --- | --- | --- | --- | --- | --- | --- |
|  |  | **20 mg** | **12 mg** | **10 mg** | **8 mg** | **6 mg** | **4 mg** | **0 mg** | **Not available** |
| 0, 0 | 80 | 76 | 1 | 4 | 1 | 0 | 0 | 0 | 2 |
| 2, 2-8 | 300 | 282 | 4 | 4 | 0 | 0 | 0 | 10 | 0 |
| 4, 10-16 | 292 | 274 | 4 | 4 | 0 | 0 | 0 | 10 | 0 |
| 6, 18-24 | 288 | 265 | 7 | 0 | 0 | 3 | 0 | 10 | 3 |
| 8, 26-32 | 264 | 240 | 7 | 2 | 0 | 3 | 0 | 10 | 2 |
| 10, 34-40 | 248 | 222 | 4 | 4 | 4 | 0 | 0 | 10 | 9 |
| 12, 42-48 | 220 | 191 | 7 | 7 | 4 | 0 | 1 | 9 | 1 |
| 14, 50-56 | 204 | 173 | 6 | 4 | 3 | 0 | 4 | 13 | 1 |
| 16, 58-64 | 168 | 140 | 3 | 3 | 4 | 0 | 4 | 13 | 1 |
| 18, 66-72 | 132 | 113 | 4 | 0 | 4 | 0 | 4 | 7 | 0 |
| 20, 74-80 | 108 | 95 | 3 | 0 | 0 | 0 | 2 | 8 | 0 |
| 22, 82-88 | 88 | 79 | 4 | 0 | 0 | 0 | 3 | 1 | 1 |
| **Total** | **2392** | **2150** | **54** | **32** | **20** | **6** | **18** | **101** | **20** |

Percentage of full doses dexamethasone administrated to the patients on protocol during the study period: 90% (2150/2392)

# **Table S14. Doses of Carfilzomib maintenance during PRO data collection**

E.g. at 2 months, the Carfilzomib doses administrated at week 2, week 4, week 6 and week 8 are displayed in the table. Therefore the “number of expected Carfilzomib doses administrated” is 4-times higher than the number of patients on protocol at each time point.

| **Month, weeks** | **Number of expected Carfilzomib doses administered** | **Carfilzomib administered** | | | | | |
| --- | --- | --- | --- | --- | --- | --- | --- |
|  |  | **56 mg/sqm** | **45 mg/sqm** | **36 mg/sqm** | **27 mg/sqm** | **0 mg/sqm** | **Not available** |
| 0, 0 | 80 |  |  |  | 78 | 0 | 0 |
| 2, 2-8 | 300 | 281 | 1 | 2 | 6 | 10 | 0 |
| 4, 10-16 | 292 | 273 | 1 | 2 | 6 | 10 | 0 |
| 6, 18-24 | 288 | 249 | 12 | 4 | 8 | 15 | 0 |
| 8, 26-32 | 264 | 228 | 17 | 4 | 3 | 12 | 0 |
| 10, 34-40 | 248 | 212 | 18 | 6 | 0 | 8 | 4 |
| 12, 42-48 | 220 | 187 | 15 | 7 | 0 | 11 | 0 |
| 14, 50-56 | 204 | 162 | 17 | 8 | 1 | 16 | 0 |
| 16, 58-64 | 168 | 130 | 15 | 8 | 0 | 15 | 0 |
| 18, 66-72 | 132 | 108 | 13 | 4 | 0 | 7 | 0 |
| 20, 74-80 | 108 | 82 | 16 | 2 | 0 | 8 | 0 |
| 22, 82-88 | 88 | 71 | 11 | 4 | 0 | 2 | 0 |
| **Total** | **2392** | **1983** | **136** | **51** | **102** | **114** | **4** |

Percentage of full doses carfilzomib administrated to the patients on protocol during the study period: 83% (1983+78)/(2392+80)
